# Fragile sites, chromosomal lesions, tandem repeats, and disease

**DOI:** 10.3389/fgene.2022.985975

**Published:** 2022-11-17

**Authors:** Mila Mirceta, Natalie Shum, Monika H. M. Schmidt, Christopher E. Pearson

**Affiliations:** ^1^ Program of Genetics and Genome Biology, The Hospital for Sick Children, The Peter Gilgan Centre for Research and Learning, Toronto, ON, Canada; ^2^ Program of Molecular Genetics, University of Toronto, Toronto, ON, Canada

**Keywords:** rare fragile sites, repeat expansions, folate sensitivity, chromatin structure, mechanisms of fragility

## Abstract

Expanded tandem repeat DNAs are associated with various unusual chromosomal lesions, despiralizations, multi-branched inter-chromosomal associations, and fragile sites. Fragile sites cytogenetically manifest as localized gaps or discontinuities in chromosome structure and are an important genetic, biological, and health-related phenomena. Common fragile sites (∼230), present in most individuals, are induced by aphidicolin and can be associated with cancer; of the 27 molecularly-mapped common sites, none are associated with a particular DNA sequence motif. Rare fragile sites ( ≳ 40 known), ≤ 5% of the population (may be as few as a single individual), can be associated with neurodevelopmental disease. All 10 molecularly-mapped folate-sensitive fragile sites, the largest category of rare fragile sites, are caused by gene-specific CGG/CCG tandem repeat expansions that are aberrantly CpG methylated and include FRAXA, FRAXE, FRAXF, FRA2A, FRA7A, FRA10A, FRA11A, FRA11B, FRA12A, and FRA16A. The minisatellite-associated rare fragile sites, FRA10B, FRA16B, can be induced by AT-rich DNA-ligands or nucleotide analogs. Despiralized lesions and multi-branched inter-chromosomal associations at the heterochromatic satellite repeats of chromosomes 1, 9, 16 are inducible by de-methylating agents like 5-azadeoxycytidine and can spontaneously arise in patients with ICF syndrome (*I*mmunodeficiency *C*entromeric instability and *F*acial anomalies) with mutations in genes regulating DNA methylation. ICF individuals have hypomethylated satellites I-III, alpha-satellites, and subtelomeric repeats. Ribosomal repeats and subtelomeric D4Z4 megasatellites/macrosatellites, are associated with chromosome location, fragility, and disease. Telomere repeats can also assume fragile sites. Dietary deficiencies of folate or vitamin B12, or drug insults are associated with megaloblastic and/or pernicious anemia, that display chromosomes with fragile sites. The recent discovery of many new tandem repeat expansion loci, with varied repeat motifs, where motif lengths can range from mono-nucleotides to megabase units, could be the molecular cause of new fragile sites, or other chromosomal lesions. This review focuses on repeat-associated fragility, covering their induction, cytogenetics, epigenetics, cell type specificity, genetic instability (repeat instability, micronuclei, deletions/rearrangements, and sister chromatid exchange), unusual heritability, disease association, and penetrance. Understanding tandem repeat-associated chromosomal fragile sites provides insight to chromosome structure, genome packaging, genetic instability, and disease.

## Introduction

**FIGURE 1:**
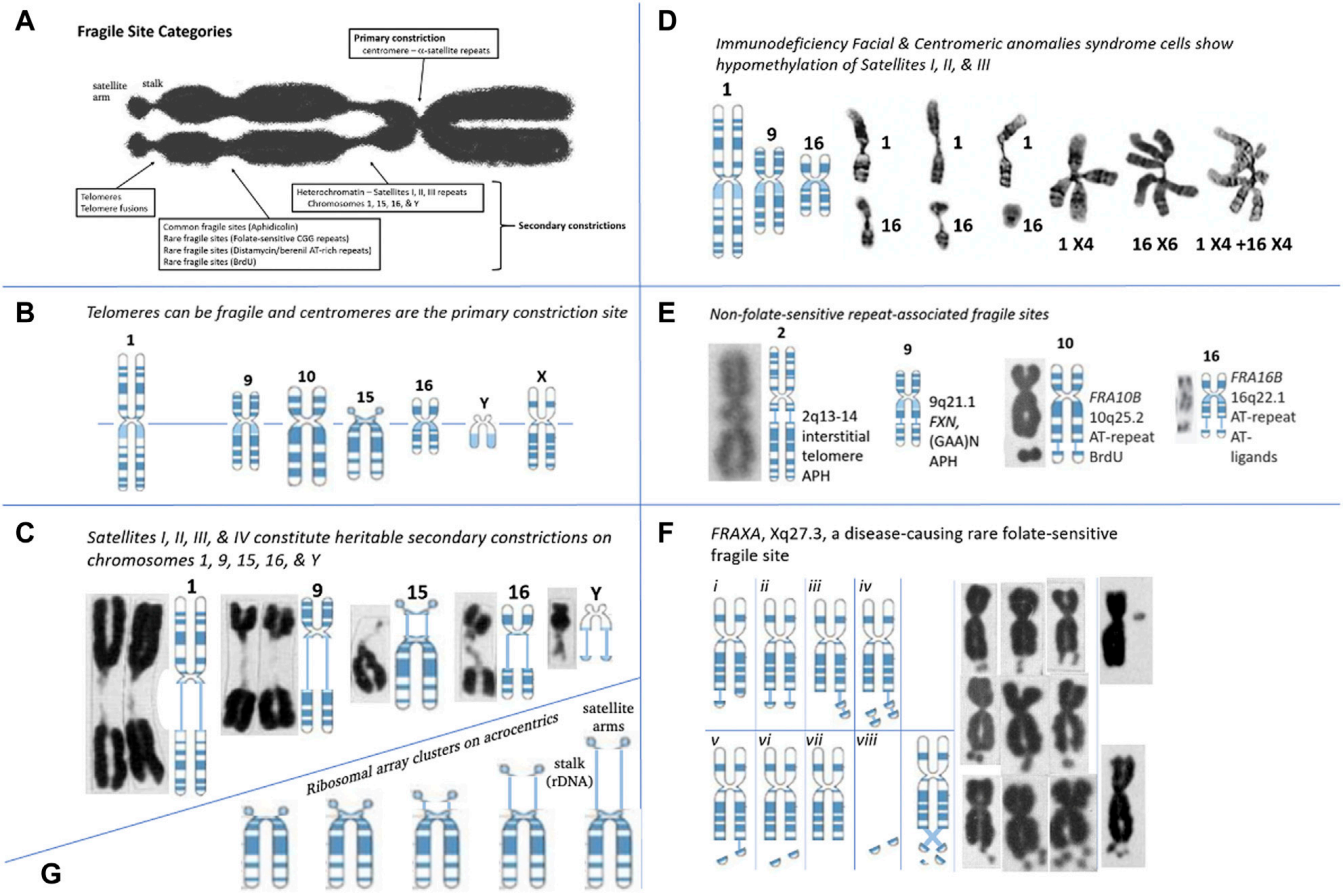
Repeat Tracts, fragile sites, and disease. **(A)** Categories of fragile site constrictions observable on human chromosomes. **(B)** Fragile sites can occur at the telomere or centromere, observed on chromosomes 1, 2, 9, 10, 15, 16, Y, and X. Telomere ends display “fragile-site like” appearances that are TRF1-dependant and APH inducible (Sfeir et al., 2009). The centromere of every chromosome is the “primary constriction,” composed of repeats. **(C)** Secondary constrictions are present on chromosomes 1, 9, 16, 15, and Y. Cytogeneticists were aware of these gaps prior to FRAXA and modeled it after the secondary constrictions (Lubs, 1969). These are composed of the classical satellite repeat DNAs, where four types (I-IV) of satellite DNA are located in the heterochromatic regions of chromosomes 1, 9, 15, 16, and Y, the total amount on these chromosomes and the proportion of the types being different (Vogt, 1990). Satellite regions of chromosomes 9 and Y, whose composition is the most complicated, and chromosome 15 is less complex, but like 9 and Y, it comprises all four types of satellite DNA. Chromosome 1 has type II satellite DNA, with the proportion of the remaining types being less. The C segment of chromosome 16 comprises only type II. The size/length of the secondary constrictions is highly-polymorphic amongst individuals, and segregates as a heritable state (Chromosomes: Guttenbach and Schmid, 1994). **(D)** In Immunodeficiency, Centromeric instability & Facial anomalies (ICF) syndrome, satellites I, II, and III of chromosomes 1, 9, and 16 are hypomethylated and show secondary constrictions within these regions. ICF syndrome was recently shown to be caused by four mutations in four genes: *ICF1/DNMT3B*, *ICF2/ZBTB24*, *ICF3/CDCA7*, and *ICF4/HELLS* (van den Boogaard et al., 2017). Satellite-containing regions on chromosomes 1, 9, and 16 are hypomethylated in individuals affected by ICF syndrome, and these show a variety of aberrant chromosomes: secondary constrictions, multibranched chromosome arms, whole arm deletions, duplications, isochromosomes, and centromeric fragility. As with fragile sites, these involved double-strand DNA breaks (Sawyer et al., 1995; Tuck-Muller et al., 2000). (Chromosomes: Tuck-Muller et al., 2000). **(E)** Many non-folate sensitive fragile sites have been mapped at repetitive regions. The interstitial telomeric repeat on chromosome 2, the AT-repeats of FRA10B and FRA16B, and the GAA repeat of *FXN* on chromosome 9 has “fragile-like” characteristics. Fragile site at 2q13-14 at an interstitial inverted head-to-head array of the telomeric repeat (TAGAGGG)54-(CCCTAA)104, a result of an ancient telomeric fusion, not “telomere healing” event (IJdo et al., 1991; Bosco and de Lange, 2012). Notably, there are other interstitial telomeric sequences (Wells et al., 1990). A “fragile-like” site has been reported at 9q21.1 in the expanded (GAA)N repeat in *FXN*, which causes Friedreich’s ataxia (Kumari et al., 2015)*. FRA10B* at 10q25.2 is induced by BrdU and mapped to an expanded ∼42 bp AT-rich minisatellite repeat (Sutherland et al., 1980; Hewett et al., 1998) (Chromosomes: Bosco and de Lange, 2012; Sutherland et al., 1980; Felbor et al., 2003). **(F)** Various presentations of the *FRAXA* site in CGG-expanded FXS patient cells (Crippa et al., 1984; Fitchett and Seabright, 1984; Savage and Fitchett, 1988): chromatid breaks/gaps, isochromatid breaks, isolated double-minutes, deleted X’s, secondary duplications (double satellite). Satellite association and variations in length of the nucleolar constriction of normal and variant human G chromosomes. (Chromosomes: Lubs, 1969). **(G)** Ribosomal DNAs can vary the length of the chromosome by varying lengths of the secondary constrictions (stalks) of the acrocentrics (Chr 13, 14, 15, 21, and 22) on which they reside (Orye, 1974; Cheung et al., 1989; Heliot et al., 1997).

The terms “fragility” and “fragile site,” coined in 1969–70, refer to unusual secondary constrictions in chromosomes, that are distinct from the primary constrictions of the centromeres (Schmid and Vischer, 1969; Magenis et al., 1970). Under specific conditions of replicative stress, they can also manifest as chromatin gaps, breaks, or failed chromatin compaction on metaphase chromosomes. Fragile sites are found across the genome, such as in the heterochromatic regions harboring classical satellite repeats on chromosomes 1, 9, 15, 16, and Y, as well as the common and rare fragile sites (Figure 1). Fragile sites can also arise at telomeres, at telomere fusions, and at other specific genetic loci. Due to their genome-wide prevalence, fragile sites have been found to be associated with genetic and genomic instability, and are extensively linked to many disease phenotypes, including neurological disorders (sections 2.1, 2.2), immunodeficiency–centromeric instability–facial anomalies (ICF) syndrome (section 2.3), and cancer progression.

The first fragile site was observed in 1965 (Dekaban, 1965), followed by the discovery of the first disease-associated fragile site at the fragile X locus (Lubs, 1969), later demonstrated to be Martin-Bell syndrome (Richards et al., 1981). This initial discovery remained largely ignored until it was serendipitously induced in specific folate-deficient culture conditions, leading to the renaming of the disease to fragile X syndrome (FXS) (Sutherland, 1977) (reviewed in (Hecht and Kaiser-McCaw, 1979). Since then, discovery of these sites at specific loci has broadened.

### Fragile site classifications

The current classifications of fragile sites fall into two categories largely based on frequency of expression and induction method: common fragile sites (CFSs) and rare fragile sites (RFSs). The Human Genome Database documents ∼90 CFSs and ∼30 RFSs that have been cytogenetically observed and documented in previous studies (reviewed by (Feng and Chakraborty, 2017).

CFSs are present in a large proportion of the population, and are induced by aphidicolin, 5-azacytidine, and bromodeoxyuridine (BrdU) (Glover et al., 1984; Yunis and Soreng, 1984; Sutherland et al., 1985b). RFSs are observed to a maximal frequency of 5% in the population (Schmid et al., 1986) and can be induced by folate deficiency/thymidylate stress, distamycin A, and BrdU (Sutherland, 1983; Hecht and Sutherland, 1984; Sutherland et al., 1985a). Detailed protocols for the detection and analysis of both CFSs and RFSs have been published recently (Bjerregaard et al., 2018). As CFSs are linked to regions of chromosomal rearrangements in cancer, this group of fragile sites has been far more extensively studied than RFSs (reviewed in (Dillon et al., 2010; Ozeri-Galai et al., 2012; Sarni and Kerem, 2016; Glover et al., 2017; Irony-Tur Sinai and Kerem, 2019; Kaushal and Freudenreich, 2019). Harnessing knowledge about CFSs could empower the field of RFSs and provide important clues as to how fragility contributes to other disease phenotypes and genetic abnormalities (i.e., repeat instability).

The current distinction between *common* and *rare* fragile sites is problematic, being based both on the conditions that induce their expression, and the frequency with which they are present in the population (Hecht, 1986; Mrasek et al., 2010). There is no clear numerical delineation between the frequency of “*common*” and “*rare*” fragile sites. Some CFSs are rare in their manifestation, suggesting they are not ubiquitously present in all individuals or might be observed at lower levels (e.g., FRA2D, FRA18B, and FRA9D are expressed in <12% of individuals) (Savelyeva et al., 2006). However, many fragile sites have been categorized as “common” when they are detected by aphidicolin induction, but have not been assessed at a population level. Distinct rare and common fragile sites have also been found to cluster together, appearing either on the same or on neighboring metaphase chromosome bands; for example, the RFS FRA11B and the APH-inducible CFS FRA11G are located at 11q23.3 (Fechter et al., 2007), and the RFS FRAXA and the APH-inducible CFS FRAXD are located at Xq27.3 and Xq27.2, respectively (Hecht and Bixenman, 1990; Sutherland and Baker, 1990) (*see* Table 1 for complete list of known clustered fragile sites). Due to this clustering, fragile sites may often be missed or misclassified, despite being independent fragility events with their own downstream consequences. Furthermore, some very common CFSs can be induced by conditions known to induce RFSs (*i.e.* folate deficiency) (Kähkönen et al., 1989; Jenkins et al., 1990; Mrasek et al., 2010). This finding demonstrates that, although certain sites may be more sensitive to specific induction methods, cytogenetic expression at a given site could be achieved with other drugs, albeit at reduced levels. Our current cytological screening methods, relying on the presence of observed metaphase chromatid breaks, may not be sensitive enough to reveal less pronounced signs of fragility at many sites. In fact, all fragile loci may be inherently sensitive to any form of replicative stress, but the ability to observe cytogenetic fragile site expression at the macro level may be uniquely influenced by their specific genomic landscape–i.e., sequence, gene expression, replication timing, among other factors. As such we propose that each CFS and RFS should also be classified on the primary induction conditions used for that locus, which may more accurately reveal similarities and differences in the characteristics and mechanisms of fragility.

**TABLE 1:** Clustered fragile sites.

Clustered fragile sites (induction method)	Chromosomal locations
FRA1M (FS) and FRA1E (APH)	1p21.3 and 1p22.2
FRA8A (FS) and FRA8B (APH) and FRA8C (APH)	8q22.1 and 8q22.1-18q22.2
FRA9A (FA) and FRA9C (BrdU)	9p21 and 9p21
FRA9B (FA) and FRA9E (APH)	9q32 and 9q32
FRA10B (BrdU) and FRA10E (APH)	10q25.2 and 10q25.2
FRA11A (FS) and FRA11H (APH)	11q13.3 and 11q13
FRA11I (FS) and FRA11C (APH)	11p15.1 and 11p15.1
FRA12D (FS) and FRA12C (BrdU) and FRA12E (APH)	12q24.13 and 12q24 and 12q24
FRA13B (BrdU) and FRA13C (APH)	13q21 and 13q21.2
FRA16B (FS and FRA16C (APH)	16q21.1 and 16q21.1
FRA16B (D-A) and FRA16C (APH)	16q22.1 and 16q22.1
FRAXD (APH) and FRAXA (FS) and FRAXE (FS) and FRAXF (FS)	Xq27.2 and Xq27.3 and Xq28 and Xq28

Induction method for each fragile site indicated in parentheses: Aph, aphidicolin; FS, folate-sensitive; BrdU, bromodeoxyuridine; DistA, distamycin A.

### Common fragile sites

The most common inducer of CFSs (∼75 sites) is aphidicolin, a deoxycytidine analogue and inhibitor of DNA polymerases α, δ, and ɛ that affects replication fork progression (Glover et al., 1984; Cheng and Kuchta, 1993). There are currently 25 molecularly mapped aphidicolin-inducible CFSs, all characterized by large AT-rich regions of DNA (reviewed in (Feng and Chakraborty, 2017) and can span a region of hundreds of kilobases to megabases of a chromosome (Mishmar et al., 1999; Zlotorynski et al., 2003; Irony-Tur Sinai and Kerem, 2019). They are frequently associated with hotspots of deletions, rearrangements, and translocations in cancer. Although the exact mechanism of aphidicolin-induced fragility is unknown, it is proposed that the induced replicative stress leads to stalling and breakage at these CFS regions due to the compounded effects of late replication, origin scarcity, concurrent transcription, and structure formation (reviewed in (Glover et al., 2017; Irony-Tur Sinai and Kerem, 2019; Kaushal and Freudenreich, 2019).

### Rare fragile sites

Thymidylate stress, caused by folate deficiency, induces the appearance of 24 of the 30 known RFSs, hereafter identified as folate-sensitive fragile sites (FSFS). To date, 10 FSFSs have been sequence-mapped to gene-specific expanded (CGG)n repeats with the most well-known site being FRAXA which occurs at *FMR1* and causes FXS. Of the remaining RFSs, three are inducible by distamycin A and three are inducible by either distamycin A or BrdU. Two of the distamycin A-inducible RFSs have been mapped to minisatellite AT-rich repeat sequences (reviewed in (Debacker and Kooy, 2007; Lukusa and Fryns, 2008). Fragility is proposed to occur when replication progression is impeded upon the binding of distamycin A (and related compounds like berenil, netropsin, Hoechst 33248, D287/170, methyl-green, and DAPI) to the minor groove of these CFS regions (Thys et al., 2015). All the currently identified RFSs, which have been cytogenetically defined and mapped and many cloned and sequenced, are highlighted in Table 2, along with known features and disease links for each. Numerous attempts to identify internal controls for diagnostic FXS by FRAXA induction revealed many sites that presented low-level (<4%) folate-sensitive fragility (reviewed in (Krawczun et al., 1991). In the proper population (disease or other) and induction systems, new rare fragile sits may be discovered. Recent discovery of new tandem repeat expansion loci could be the molecular cause of new, as yet to be observed fragile sites or chromosomal lesions (Giannuzzi et al., 2021; Altemose et al., 2022; Ebler et al., 2022; Gershman et al., 2022; Hoyt et al., 2022; Nurk et al., 2022; Talbert and Henikoff, 2022; Vollger et al., 2022; Wang et al., 2022).

**TABLE 2:** Rare fragile sites (folate, distamycin A, and BrdU).

Fragile site (name and location), induction method, mapped sequence (if known) and allele sizes	Other characteristics	Linked diseases and publications
FRAXA **-** Xq27.3	• Expression of *FMR1* is silenced when expanded and methylated	• Fragile X syndrome: inherited ID (Sutherland, 1977)
	• Expression of *FMR1* is enhanced up to 10-fold for premutation expansion and no methylation	• Mapping of sequence: (Kremer et al., 1991; Oberlé et al., 1991; Verkerk et al., 1991; Yu et al., 1991)
Folate deficiency	• presence of AGG-anchoring trinucleotides doesn’t affect fragile site expression (Zhong et al., 1995)	• Deletions and instability at Xq27 observed in Fragile X: (Gedeon et al., 1992; Wöhrle et al., 1992; Tarleton et al., 1993; Gu et al., 1994; Trottier et al., 1994; Hirst et al., 1995; Lugenbeel et al., 1995)
(CGG)n-N; *FMR1* gene	• FS can be detected in pre-mutation expansion cells, as well as in unaffected females, where expression can vary	• Fragile X Associated Tremor Ataxia (FXTAS) (Hagerman and Hagerman, 2001)
(CGG)6–52; Non-affected	• Adjacent mutation hotspot	• Fragile X-associated Primary Ovarian Insufficiency (FXPOI): (Allingham-Hawkins et al., 1999; Murray, 2000)
(CGG)59–230; Premutation	• Unusual chromatin compaction	• Autism oFull mutation: (Brown et al., 1982)
(CGG)230–2000; Full-mutation		• Pre-mutation: (Tassone et al., 2000; Hagerman and Hagerman, 2002; Aziz et al., 2003)
Aberrant CpG methylation		• *FMR1* locus is linked with hypermutations, deletions, duplications, CNVs, *etc.* → all mutation types causing Fragile X syndrome documented at: http://www.hgmd.cf.ac.uk/ac/gene.php?gene=FMR1
FRAXE **-** Xq28	• Expression of *FMR2* is silenced when expanded	• Observed by: Sutherland and Baker, 1992
Folate deficiency	• missense mutations in highly conserved *FMR2* sites are linked to autism	• Mapping: (Knight et al., 1993)
(CGG)n-N; *FMR2/AFF2* gene	• ∼600 kb distal to FRAXA	• X-linked ID: (Knight et al., 1993, 1994)
(CGG)4–39; non-affected	• FS can be detected in pre-mutation expansion cells, as well as in unaffected females, where expression can vary	• *FMR2* gene identification: (Gecz et al., 1996)
(CGG)31–61; premutation		• Deletions, missense mutations and duplications of *AFF2* gene linked to ID and autism: (Gecz et al., 1996; Moore et al., 1999; Probst et al., 2007; Whibley et al., 2010; Cavani et al., 2011; Stettner et al., 2011; Mondal et al., 2012)
(CGG)200–900; full mutation		
Aberrant CpG methylation		
FRAXF **-** Xq28	• Expansion silences *FAM11A* expression	• Observed by: (Hirst et al., 1993)
Folate deficiency	• 5-azadeoxycytidine reactivates *FAM11A* transcription = methylation important in silencing	• Mapping: (Parrish et al., 1994; Ritchie et al., 1994)
(CGG)n-N; *FAM11A* gene	• ∼600 kb distal to FRAXE	• Gene characterization: (Shaw et al., 2002)
(CGG)7–40; non-affected	• FS detected in pre-mutation expansion cells and seemingly unaffected females (expression can vary)	• Ritchie et al., 1994: suggests link to retardation where a male with developmental delay had 900 methylated repeats
(CGG)306–1008; full mutation		• Parrish et al., 1994: several related individuals expressing fragile site but no ID and several probands expressing fragile site with ID, hence disease link is questionable
Aberrant CpG methylation		
FRA1M **-** 1p21.3	Not mapped	• Mentioned in review: (Lukusa and Fryns, 2008)
Folate deficiency		
FRA2A - 2q11.2	• silenced *AFF3* gene due to expanded hypermethylation of CGG in conserved, brain-active alternative promoter	• Mapping and link to three families w/wide spectrum of neurodevelopmental phenotypes; mostly motor and language delays of varying degrees (Metsu et al., 2014b)
folate deficiency	• *AFF2/FMR2* is X-linked homolog of *AFF3*	• FS in schizophrenia cells: (Chen et al., 1998)
(CGG)n-N; *AFF3* gene	• expanded CGG in *AFF3* can form G-quadruplexes	• Severe multi-system disorder in patient with *de novo* microdeletion of only *AFF3* (Steichen-Gersdorf et al., 2008)
(CGG)5–18; non-affected	• *AFF3* can bind G-quadruplexes, so could autoregulate itself @ promoter	• Phenotype difference between expansion and deletion of *AFF3* could be due to the expansion causing gene silencing later in development or it affecting only the brain-specific promoter, causing a milder, non-systemic phenotype
(CGG)∼100- premutation		
(CGG)>300; full mutation		
Aberrant CpG methylation		
FRA2B **-** 2q13	Not mapped	• Mentioned in review: (Lukusa and Fryns, 2008)
Folate deficiency		
FRA2K **-** 2q22.3	Not mapped	• Mentioned in review: (Lukusa and Fryns, 2008)
Folate deficiency		
FRA2L **-** 2p11.2	Not mapped	• Mentioned in review: (Lukusa and Fryns, 2008)
Folate deficiency		
FRA5G - 5q35	Not mapped	• FRA5G FS observed in patient with ID and an unaffected brother (Howell et al., 1990)
Folate deficiency		
FRA6A **-** 6p23	Not mapped	• Linkage of 6p23 region to schizophrenia (Olavesen et al., 1995)
Folate deficiency		
FRA7A - 7p11.2	• expansion within 5′ intron of *ZNF713*, a zinc-finger protein and a regulator of transcription	• Mapping and autism spectrum disorder link: (Metsu et al., 2014a)
Folate deficiency	• *SEPT14,* a nearby gene could also be involved but its expression was undetectable	
(CGG)n-N; *ZNF713* gene	• Reduced transcription of *ZNF713* with expansion	
(CGG)5–22; non-affected		
(CGG)42–85; premutation		
(CGG)>450; full mutation		
Aberrant CpG methylation		
FRA8A - 8q22.3	Not mapped	• Mentioned in review: (Lukusa and Fryns, 2008)
Folate deficiency		
FRA8E - 8q24.1	• involved in various chr rearrangements associated w/Langer-Giedion syndrome but most FRA8E carriers are healthy subjects	• (Bühler and Malik, 1984; Takahashi et al., 1988; Lüdecke et al., 1991; Hou et al., 1995)
Distamycin A		• Cloning of region near *EXT1* gene and HPV16 DNA integration site (Hori et al., 1998)
FRA9A **-** 9p21	Caused by (GGGGCC)n expansion in *C9orf72* gene (Lab of C.E. Pearson, in preparation)	• Observed by: (Sutherland et al., 1983; Kähkönen, 1988)
Folate deficiency		• Most common rare FSFS in Finnish population (Kähkönen, 1988)
		• Not observed in Japanese population (Takahashi et al., 1988)
		• FS in schizophrenia cells: (Garofalo et al., 1993, 1992)
FRA9B **-** 9q32	Not mapped	• Observed in: (Sutherland, 1982; Petit et al., 1986)
Folate deficiency		
FRA10A - 10q23.3	• single, imperfect but polymorphic CGG repeat in CpG island of 5′ UTR of *FRA10AC1*, a novel ubiquitously expressed nuclear protein	• Mapping: (Sarafidou et al., 2004)
Folate deficiency	• transcriptional silencing of 1 allele in expansion carriers (likely FRA10A FS-expressing allele)	• In heterozygous state: expansion is likely benign; no homozygotes known
(CGG)n-N; *FRA10AC1* gene	• most prevalent among the rare autosomal folate-sensitive fragile sites in human genome	• ID link: (Petit et al., 1986; Mavrou et al., 1991)
(CGG)8–14; non-affected		• Highest rate of rearrangements/deletions in prostate tumors occurs at 10q23-q24: (Lacombe et al., 1996)
(CGG)>200; full mutation		• Frequent lung cancer deletions at 10q23-26: (Kim et al., 1998)
Aberrant CpG methylation		• (Villa et al., 1997): showed that *de novo* telomeric repeats occur at the FRA10A break
FRA10B - 10q25.2	• has varying minisatellite repeats of diff lengths (has 42-bp consensus sequence)	• no disease link – homozygotes for both FRA10B and FRA16B have been identified as normal (Sutherland, 1981)
distamycin A or BrdU induced	• fragile site is present when repeat is > 5 kb	• (Scheres and Hustinx, 1980; Sutherland et al., 1980)
AT-rich (91%) expanded ∼42-bp repeat unit		• (Hewett et al., 1998; Handt et al., 2000; Schwartz et al., 2006)
FRA11A **-** 11q13.1	• expansion in 5′ UTR of *C11orf80* gene causing fragile site and transcriptional silencing	• Mapping and ID (in 1 of 5 individuals w/FSFS within same family) (Debacker et al., 2007)
Folate deficiency	• unknown function with no homology to other known genes	• Other ID links: (Sutherland, 1979; Sutherland, 1982; Hecht and Sutherland, 1985; Smeets et al., 1985)
(CGG)n-N; *C11orf80* gene		
(CGG)6–8; non-affected		
(CGG)>500; full mutation		
Aberrant CpG methylation		
FRA11B **-** 11q23.3	• located in the 5′ UTR of the *CBL2* proto-oncogene	• Associated with chromosome deletion characteristic of Jacobsen’s syndrome (ID/facial abnormalities) where portions of long arm of chromosome 11 is lost (Voullaire et al., 1987; Jones et al., 1994, 1995; Michaelis et al., 1998)
Folate deficiency	• 1^st^ report of a direct link between a fragile site and chromosome breakage *in vivo*. Mother had an expansion and fragile site but her child inherited deletion with the breakpoint in the fragile site region, stabilized by the *de novo* addition of a telomere (Jones et al., 1994)	• Mapping: (Jones et al., 1995, 1994)
CGG)n-N; *CBL2* gene	• FS typically observed in unaffected parents of non-FS-expressing Jacobsen syndrome children (11q deletion)	• First observed: (Hecht and Sutherland, 1985)
(CGG)8–14; non-affected		
(CGG)85–100; premutation		
(CGG)100->1000; full mutation		
Aberrant CpG methylation		
FRA11I - 11p15.1	Not mapped	• Mentioned in review: (Lukusa and Fryns, 2008)
Distamycin A		
FRA12A **-** 12q13.1	• Methylated repeat expansion in promoter of *DIP2B* gene	• Mapping and ID due to decreased expression (Winnepenninckx et al., 2007)
Folate deficiency	• WT *DIP2B* is likely involved in DNA methylation processes	• (Giraud et al., 1976): identified chromosomal breakage point in 12q13 in male with ID and multiple congenital anomalies
(CGG)n-N; *DIP2B* gene	• premutation carriers: have increased gene expression due to lack of methylation but still have fragile site expression (reduced)	• Retardation: (Smeets et al., 1985)
(CGG)6–23; non-affected		• Proband with MR; mother and grandmother unaffected: (Berg et al., 2000)
(CGG)∼130–200; premutation		
(CGG)>900; full mutation		
Aberrant CpG methylation		
FRA12C **-** 12q24	• Not mapped	• Mentioned in review: (Lukusa and Fryns, 2008)
BrdU induction and folate deficiency		
FRA12D **-** 12q24.13	Not mapped	• segregates in FX families (Amarose et al., 1987; Barletta et al., 1991)
Folate deficiency		• Observed in: (Sutherland and Baker, 1993)
FRA16A **-** 16p12.3	• (Nancarrow et al., 1994): observed 72 repeat CGG unaffected individual without FS expression	• Mapping: (Nancarrow et al., 1994)
Folate deficiency	• expanded repeat is adjacent to a CpG island that is methylated in fragile site-expressing individuals	• Baratela-Scott Syndrome link: (LaCroix et al., 2019)- linked repeat to autosomal recessive disease, Baratela-Scott Syndrome -this is an important paper, as this fragile site was previously identified as not being associated with disease when inherited as a heterozygous CGG expansion, but when homozygous displays disease. They also report deletions and other mutations leading to pathogenic variants in 1 allele of *XYLT1* in these patients with expansions. Other forms for other fragile sites may arise where either both alleles are expanded, or one is expanded, and the other allele is mutant elsewhere in the associated gene
(CGG)n-N; *XYLT1* gene	• individuals who do not express the fragile site do not have DNA methylation	
(CGG)9–20; non-affected	• Transcriptional silencing due to expanded methylated alleles (LaCroix et al., 2019)	
(CGG)300–2500; full mutation		
Aberrant CpG methylation		
FRA16B - 16q22.1	• As many as 2000 repeats cause FRA16B expression (7–12 copies in WT allele)	• First observed with Mendelian inheritance: (Magenis et al., 1970)
distamycin A or BrdU induced	• first report of mini-satellite repeat expansion	• (Sutherland et al., 1984; Yu et al., 1997; Hocking et al., 1999; Hsu and Wang, 2002)
33-bp AT-rich repeat; or 35-bp AT-rich repeat;	• strongly excludes nucleosome formation only in presence of distamycin	• No disease link (homozygous and heterozygous individuals)
		• FRA16B has been mapped to a 33-base pair AT-rich minisatellite repeat (Yu et al., 1997) as well as a 35-base pair repeat (Yamauchi et al., 2000)
		• FRA16B is the most common of the rare fragile sites, expressed in 5% of the European population (Felbor et al., 2003)
FRA16E - 16p12.1	Not mapped	• Mentioned in review: (Lukusa and Fryns, 2008)
Distamycin A		• many deletions known to occur in this region (*ex*. 16p21 deletion syndrome) and be associated w/developmental delay
FRA17A - 17p12	Not mapped	• Mentioned in review: (Lukusa and Fryns, 2008)
Distamycin A/BrdU		
FRA19B - 19p13	Not mapped	• Mentioned in review: (Lukusa and Fryns, 2008)
Folate deficiency		
FRA20A 20p11.23	Not mapped	• Mentioned in review: (Lukusa and Fryns, 2008)
Folate deficiency		
FRA22A - 22q13	Not mapped	• associated with ID (Webb and Thake, 1984)
Folate deficiency		

Size ranges of repeats for some of the mapped fragile sites are reported estimates, which in some cases are limited by the small number of affected and reported families. Other fragile sites that presented low-level (<4%) folate-sensitive fragility have been documented are covered in detail elsewhere (Krawczun et al., 1991).

### Spontaneous fragile site expression

Spontaneous fragile sites occur without the need for induction at chromosomal locations distinct from either the common or rare fragile sites. These spontaneous sites can be expressed at unusually high levels, from 80 to 100% of the population, compared to the 4–30% for most fragile sites (Dar et al., 1995; Karadeniz et al., 2003; Zamani et al., 2007). The nature of the molecular cause (sequence, epigenetic, or other) of most of these spontaneous fragile sites is not known and warrants further investigation. Examples include the secondary constrictions on chromosomes 1, 9, 16, and Y, as well as FRA1R/1q41 and FRA16B/16q22. It is possible that these spontaneous sites are due to repetitive sequences, as the spontaneous FRA16B has been mapped to a 33-base pair (bp) AT-rich minisatellite repeat (Yu et al., 1997) as well as a 35 bp repeat (Yamauchi et al., 2000). FRA16B is the most common of the RFSs, expressed in 5% of the European population (Felbor et al., 2003). Other spontaneous fragile sites have been localized to intra-chromosomal telomere tracts (Musio et al., 1996), which are frequent polymorphisms of heterochromatin without known functional or phenotypic effect. The length of the chromosomal gaps or despiralized regions can vary widely between individuals, is considered to be hereditary, and due to the highly variable lengths of the satellite tracts (Craig-Holmes and Shaw, 1971; Yunis and Yasmineh, 1971; Craig-Holmes et al., 1975, 1973; McKenzie and Lubs, 1973; Podugolnikova and Korostelev, 1980). These spontaneous, heritable fragile sites often map to loci known to be prone to structural variations including microdeletions, microduplications, and copy number variations (CNVs) (Zamani et al., 2007; Szafranski et al., 2010; Gillentine and Schaaf, 2015). Viral integration can also be a driving factor for these spontaneous sites (O’Neill and Miles, 1969; Peat and Stanley, 1986) (reviewed in (Fortunato and Spector, 2003). Interestingly, chromosomal integration of tandem repeats of foreign DNA can lead to fragile site expression, further supporting the possibility that repeat tracts underlie spontaneously expressed fragile sites (Ragland et al., 2008; Jacome and Fernandez-Capetillo, 2011; Irony-Tur Sinai et al., 2019).

### Mapping fragile sites

**FIGURE 2:**
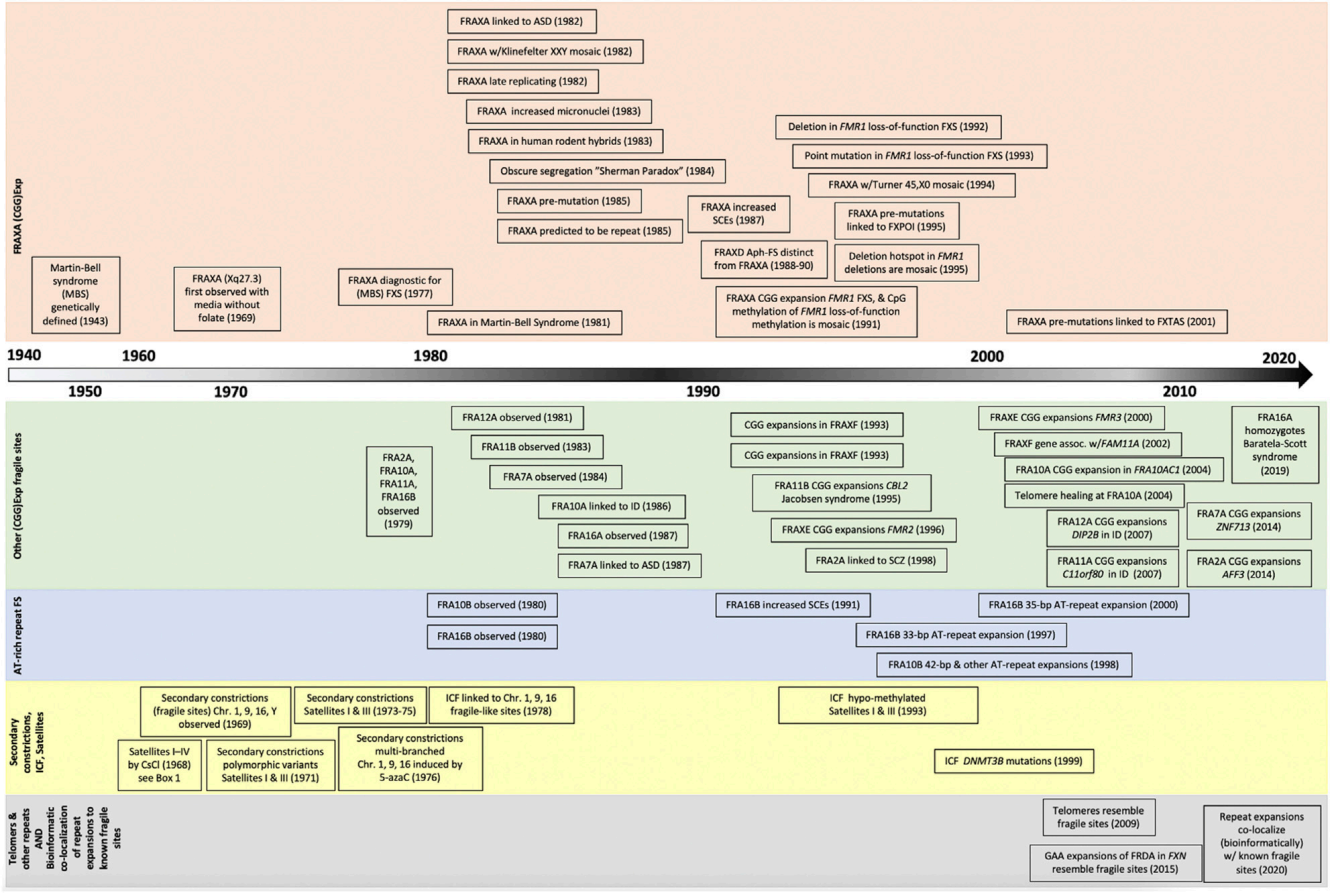
Timeline of discovery for tandem repeat expansions and chromosomal lesions. Increased awareness and improved methods of detection have fostered the identification of dozens of fragile site in the past two decades, with new bioinformatic techniques poised to launch an new era of fragile site discoveries.

As mentioned, many CFSs and RFSs have been observed cytogenetically; however, only a handful have been molecularly mapped to specific genomic locations, or specific sequences. Mapping fragile sites is an investment, as the efforts from initial cytogenetic observation, to molecular mapping, to gene identification and epigenetic modifications, can be considerable and span years (Figure 2). Mapping of fragile sites dates to the 1980s, where R-banding was performed, and the general chromosomal site of the observed break was reported. This technique was utilized to determine the chromosomal location of the DAPI-inducible CFS FRA1H (Pelliccia and Rocchi, 1986), providing the basis for further, more detailed mapping. Using yeast artificial chromosomes (YACs), bacterial artificial chromosomes (BACs), and cosmid clones that span the region of the identified cytogenetic location, physical mapping and fluorescence *in situ* hybridization (FISH) experiments allowed for further characterization of the genomic location of these fragile sites, albeit still at a low resolution. Some examples of both CFSs and RFSs that were mapped in such a manner include FRAXA (Kremer et al., 1991; Verkerk et al., 1991), FRA11B (Jones et al., 1994), FRA3B (Boldog et al., 1994), FRA16D (Paige et al., 2000), FRAXB (Arlt et al., 2002), and FRA7B (Bosco et al., 2010). Clustered fragile sites (touched upon in section 1.1) can require finer mapping in order to be distinguished. Higher resolution mapping has been performed with the use of multi-colour FISH combined with the availability of sequence databases and programs. With this method, an initial large region spanning the cytogenetic location of the fragile site is covered with BAC probes labelled with different colors. Increasingly finer mapping is conducted with contiguous multi-colored BAC probes spanning smaller and smaller lengths across the break point until an exact breakage boundary can be determined. The specific sequence of this region along with the encompassing genes are then identified through programs such as RepeatMasker and through human genome sequence databases (Hormozian et al., 2007; Zheglo et al., 2019). The identification of these specific fragile site-associated genes can initiate further studies on the role of fragile sites in human genetic diseases and cancer.

**FIGURE 3:**
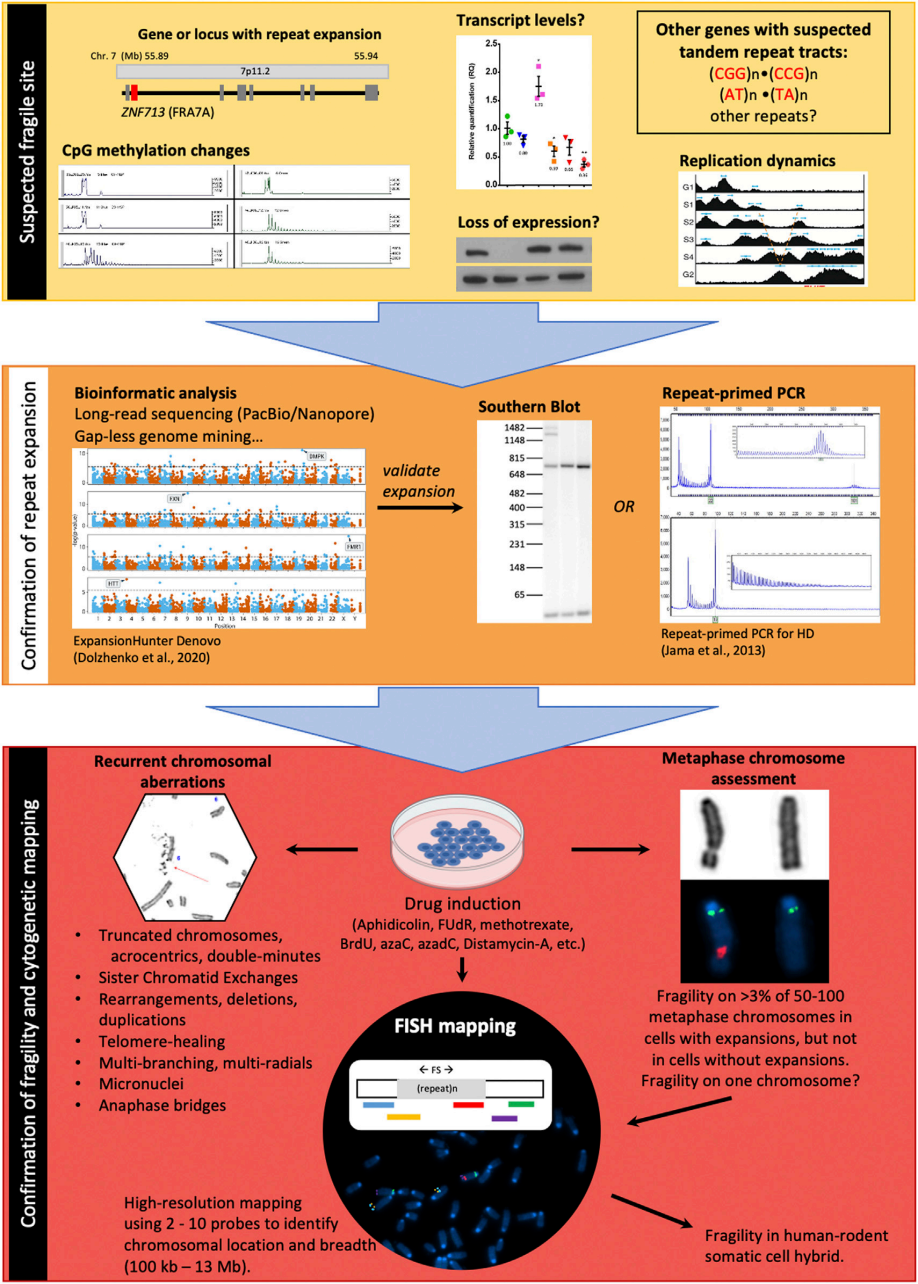
Strategies to identify and map a fragile site. *Yellow segment–*Repeat-associated FS breakpoints may be suspected based upon various genetic and epigenetic landmarks in normal cells. Large genes, changes in CpG methylation patterns on one chromosome, loss of expression (transcript or protein), a V-shaped replication timing pattern, and the presence of tandem repeat sequences (CGG)n, (GGGGCC)n, and (AT)n are all pre-disposing factors for FSs. *Orange segment–*confirmation of a repeat expansion at the suspected FS can first be carried out by bioinformatic analysis of sequenced reads, followed by validation *via* Southern blot (gold standard) or repeat-primed PCR. *Red segment–*The final step of FS localization requires drug treatment to induce expression of the FS, followed by characterization of metaphase spreads for fragile site hallmarks and FISH-based mapping of the FS using two or more coloured FISH probes.

As in the case of CFSs, the mechanisms and common sequence motifs that are shared between these regions are merely beginning to be elucidated, having previously been limited by early cytogenetic methods used to fine map fragile regions (i.e., physical mapping and FISH). The onset of bioinformatic methods and databases in recent years provides the potential to simultaneously identify many regions prone to fragility, making them strong candidates for further analysis. Prada and Laissue (2014) used bioinformatic methods to identify chromosomal rearrangements of the X chromosome in 13 different mammalian species (Prada and Laissue, 2014). They identified fragile sites previously associated with the human X chromosome (FRAXA, B, C, D, E, and F), and were also able to determine fragile sites that are conserved between mammalian species, implying that these regions could have functional roles. Their work characterizing the X chromosome provides exciting new avenues for expansion to the rest of the genome and in identifying novel important regions of fragility. Ji et al. (2020) provided a genome-wide mapping of CFSs by using the previous knowledge that most CFSs undergo mitotic DNA synthesis (MiDAS); by sequencing the nascent DNA in mitotic cells treated with aphidicolin, novel aphidicolin-inducible CFSs were able to be uncovered (Ji et al., 2020). The methods of mapping the molecular cause of a fragile site are outlined in Figure 3, and could include CNVs in variable number tandem repeats, identified by bioinformatic tools such as ExpansionHunter Denovo (Garg et al., 2020; Trost et al., 2020). Overall, the current improvements in methodology and technology allowing for more detailed and quicker discovery of CFSs and RFSs provides the potential to advance the understanding of these fragile regions. Further studies on common genomic features such as sequence, epigenetic landscapes, and expression profiles would allow for the development of more accurate automated programs for the discovery of novel fragile sites. Moreover, revised “gapless” reference genomes should further facilitate the suspected association of tandem repeats with fragile sites, speeding the mapping process (Figure 3).

## Fragile sites and repeats

A variety of repeat sequences–including telomeric, centromeric, classical satellite repeats I, II, and III, and various disease-related repeats–have been mapped as fragile sites, chromosomal lesions, or chromatin aberrations (Warburton et al., 1996; Sfeir et al., 2009; Bosco and de Lange, 2012; Black and Giunta, 2018) (Figure 2, *see* also Box 1). Unlike CFSs, which arise at genomic regions with no clear sequence motif, all mapped disease-associated RFSs arise at repeat sequence motifs, including the CGG expansion-associated sites (FRAXA, FRAXE, FRAXF, *et cetera*). In the past decade, there has been a steady discovery of new folate-sensitive disease-associated fragile sites, including expanded CGG tracts associated with FRA2A (Metsu et al., 2014b), and FRA7A (Metsu et al., 2014a). Most recently, the rare autosomal-recessive Baratela-Scott syndrome was reported to be associated with the FRA16A CGG expansion in the homozygous state (LaCroix et al., 2019). This site was originally reported 25 years ago as benign when heterozygous (Nancarrow et al., 1994). It should be noted that some homozygously expressed fragile sites have not been associated with disease (FRA10B, FRA16B, and FRA17A) (Berg et al., 1969; Sutherland, 1981; Voiculescu et al., 1991; Felbor et al., 2003). Thus, the phenotypic impact of a fragile site must be considered as other genetic variations.

Box 1 **Satellite terminology**

Acrocentric or satellited chromosomes, where the “satellited” chromosomal arm is telomeric to a secondary constriction -the centromere (Ferguson-Smith and Handmaker, 1961). The compact heterochromatic region, known as the stalk, between the centromere and the satellite arm is repetitive satellites and rDNA clusters. Satellited chromosomes were observed to form inter-chromosomal satellite associations (Ferguson-Smith and Handmaker, 1961). Similar to the multi-branched chromosomes in ICF syndrome (*see* section 2.3), satellite associations are genetically inherited (Ferguson-Smith and Handmaker, 1961). The term “satellite DNA” was first named where density separation (isopycnic gradients on CsCl or Ag+-Cs_2_S0_4_) of genomic DNAs were found to resolve as multiple distinct bands; a major band and numerous “satellite DNA” bands (Kit, 1961). The density difference between bands was subsequently found to be due to the limited and tandem repetitive nature of the DNA sequences in the bands (Jones and Corneo, 1971; Jones et al., 1973; Jones et al., 1974; Gosden et al., 1975; Frommer et al., 1982; Prosser et al., 1986), and hence their being termed “satellite repeats” (with units of 5–171 bp), microsatellites (with motifs of 1–4 bp), minisatellites (with motifs of 5–64 bp), megasatellites/macrosatellites (motifs of up to several hundred kb), and tandem gene amplifications. Human DNA contains at least four defined isopycnic density bands: satellite I (1.687 g/ml), satellite II (1.693 g/ml), satellite III (1.696 g/ml), and satellite IV (1.700 g/ml) (Corneo et al., 1968; Corneo et al., 1970; Corneo et al., 1972). These constitute respectively ∼0.5, ∼2.0, ∼1.5, and ∼2.0% of the total genomic DNA. Interestingly, the DNA constituting the secondary constriction of some satellited chromosomes, turns out to be due to repeat expansions, as in FRAXA, ICF, *etc.* New sequencing and bioinformatic tools are only beginning to harness a full appreciation of these tandem repeats and their relationship to chromosome structure (Cechova, 2020; Liehr, 2021; Suzuki and Morishita, 2021; Thakur et al., 2021; Altemose, 2022; Altemose et al., 2022; Cechova and Miga, 2022; Gall-Duncan et al., 2022; Hoyt et al., 2022; Nurk et al., 2022). This nomenclature, while not comprehensive, lacks clear boundaries. It was recently suggested so as to avoid confusion, especially with the ever-increasing number of TRs with units of almost any length, to use the term “tandem repeat (TR), with a motif of X nucleotides” (Gall-Duncan et al., 2022).

**FIGURE 4:**
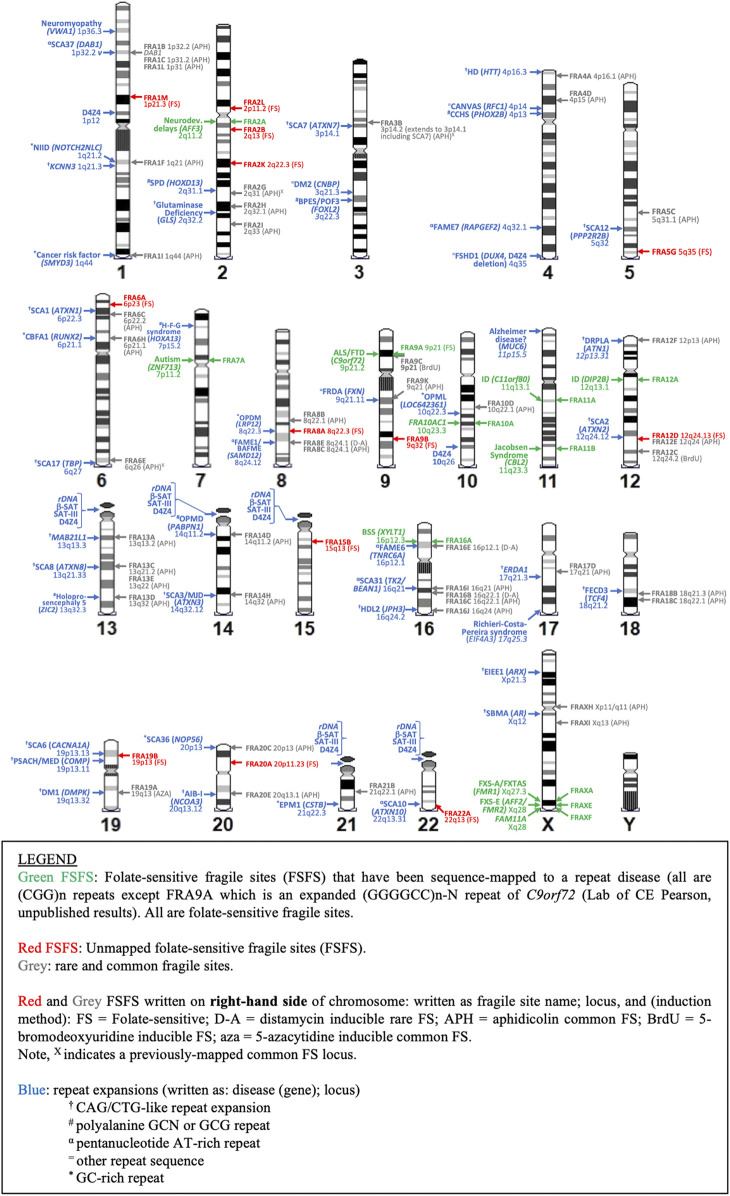
Karyotypic ideogram of repeat expansions and fragile site locations. Ideogram shows the mapping of all disease and non-disease repeat expansions (blue; on left side of chromosome) compared to all folate sensitive fragile sites (FSFS) (red and green; right side) and selected rare and common FS near disease loci (gray; right side).

Technological advances are driving the discovery of additional tandem repeats and disease-linked CGG repeat expansions (Ishiura et al., 2019, 2018; Sone et al., 2019). These repeats could be the molecular cause of novel undiscovered fragile sites and warrant further investigation. Our recent work identified over 2500 repeat motifs significantly enriched in the genomes of autistic patients (Trost et al., 2020). Many of these repeats colocalized to cytogenetically observed, but not molecularly mapped FSFSs (Trost et al., 2020). Using epigenetic-based methodologies, others have also computationally identified abnormally hypermethylated CpG-rich tandem repeat loci colocalizing to unmapped FSFSs (Garg et al., 2020). It is not clear whether these epigenetically mapped TRs actually require aberrant CpG methylation for expression (Garg et al., 2020), as other cytogenetically mapped FSFS do not require methylation with repeat expansion being sufficient for expression (Smeets et al., 1995; Perroni et al., 1996; Winnepenninckx et al., 2007). Other repeat sequences could also manifest as fragile sites under the correct inducing conditions, as the unique conditions necessary to induce fragility at different repeat sequences may not yet be understood. Additionally, there are several repeat expansion disease loci in regions not yet associated with fragile sites but may show fragility only in currently uncharacterized patient populations. Figure 4 compares the cytogenetic location of all known repeat expansions against neighboring common and rare fragile sites previously identified in the literature. In the next section we review the various types of repeats, associated disease, and fragility.

### (CGG)n repeat expansions cause folate-sensitive fragile sites: FRAXA and other loci

Ten FSFSs have been molecularly mapped to gene-specific expanded (CGG)n repeats. These sites include FRAXA (at *FMR1* - causing FXS, FRAXE, FRAXF, FRA2A, FRA7A, FRA10A, FRA11A, FRA11B, FRA12A, and FRA16A (details and relevant citations in Table 2). While it is clear that an expansion is required for fragile site expression, there is only a mild effect of larger expansions on fragility (Rousseau et al., 1994), supporting the importance of the presence of a repeat expansion over its size. Each of these sites shows aberrant CpG methylation both upstream of and at the repeat, which is associated with loss of transcription of the expanded allele. Most of these 10 characterized FSFSs have been associated with some form of neurological disease, with 16 other FSFSs remaining uncharacterized with respect to sequence and disease association. As such, it is possible that some other non-CGG repeat may be involved with the uncharacterized FSFS. Moreover, there may be additional undiscovered FSFSs for which the disease-causing mutation may be a GC-rich repeat motif.

**FIGURE 5:**
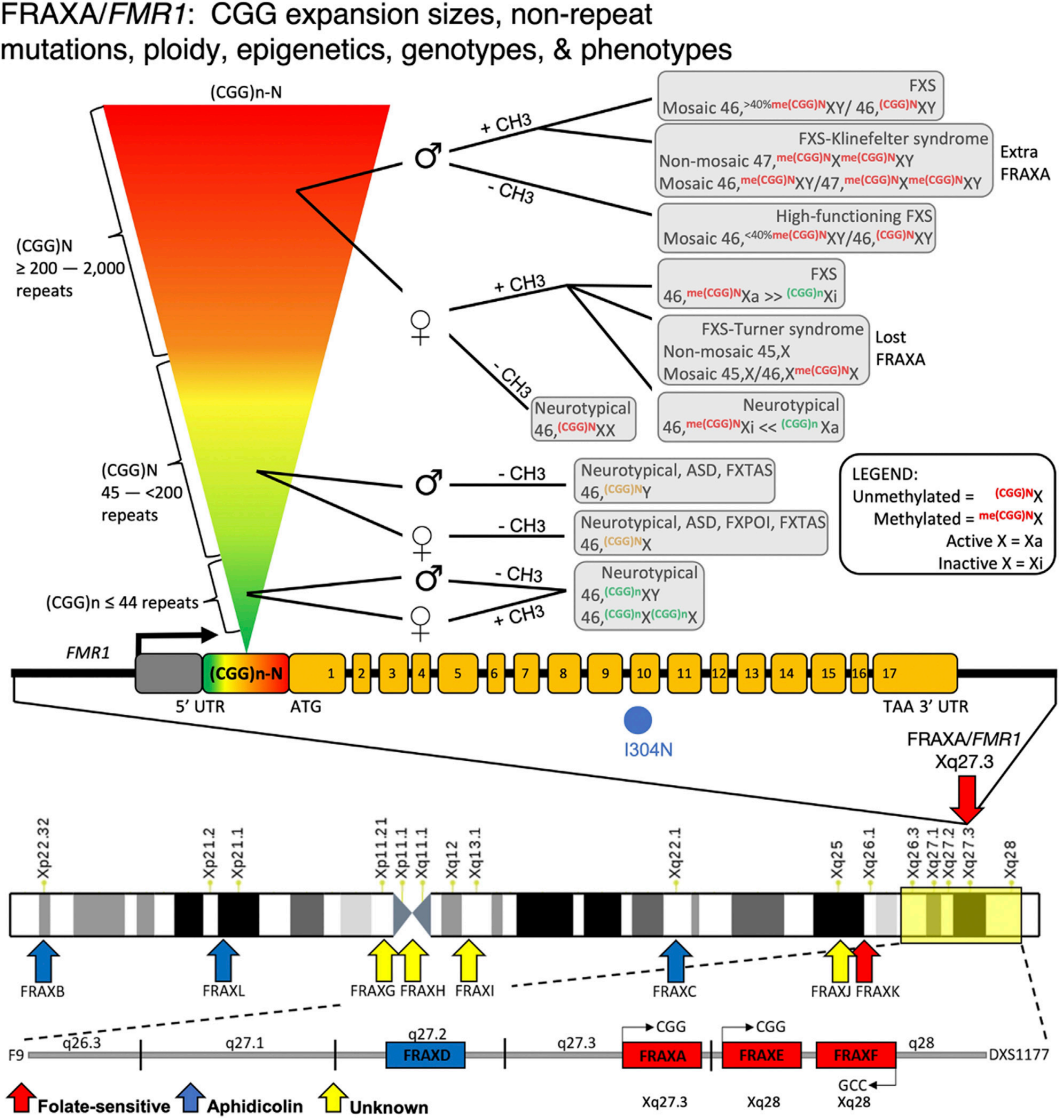
*FMR1/*FRAXA numerous mutations, genotypes, and phenotypes. Disease mosaicism reported at the *FMR1* (CGG)n repeat locus, influenced by repeat size (rainbow inverted triangle showing increasing repeat size), DNA methylation of the repeat expansion ( ± CH3), and sex of patient (♂ = male; ♀ = female). Individuals with <44 repeats, regardless of sex, are neurotypical. Those with repeats between 45 and 200 can have Fragile X-Associated Tremor/Ataxia Syndrome (FXTAS) or autism if male with unmethylated repeat. If female, these individuals have unmethylated alleles that are likely neurotypical or have Fragile X primary ovarian insufficiency (FXPOI). With expansions >200 repeats, the disease spectrum becomes more variable. In females, X-inactivation affects disease outcome. Due to the presence of two X alleles in females and random X-inactivation, DNA methylation effects can vary substantially between individuals depending on which allele is X-inactive (Xi) compared to active (Xa). This is also influenced by which tissues are affected and what degree of mosaicism in X-inactivation exists in the patient. If allele is methylated, females can show FXS or Turner syndrome mosaicism based on which allele is Xi vs. Xa. Males with unmethylated expanded alleles are high functioning FXS. Methylated individuals can have FXS or FXS with mosaic Klinefelter syndrome. Many deletions within this *FMR1* promoter region have been reported, causing *FMR1* silencing and FXS syndrome.

The most extensively studied fragile site, FRAXA, provides a complex picture of the mutational and disease heterogeneity that can arise from a single fragile site. Depending on expansion size, methylation status, and sex, different diseases manifest within patients, many of whom have vastly different symptomatic features (Figure 5) (reviewed in (Nichol Edamura and Pearson, 2005; Lozano et al., 2014; Hagerman et al., 2018). Various mutation forms and epimutations at *FMR1* were identified to be the cause of a broad spectrum of clinical presentations, including FXS, autism, fragile X-associated ataxia (FXTAS), premature ovarian failure/insufficiency (FXPOI), attention-deficit disorder, learning disabilities, as well as psychologic, endocrine, autoimmune, and metabolic disorders (Hagerman et al., 2018). Interestingly, this complexity in disease manifestation at the FRAXA locus has only recently become apparent, some 75 years after the initial reports of FXS as Martin-Bell syndrome (Martin and Bell, 1943). Given such a complex etiology at this particular locus, enormous unrecognized and unexplored complexity may exist at other fragile sites.

### AT-rich repeats at CFSs and RFSs

The sequences of all CFSs mapped thus far exhibit a strong skew towards AT-rich regions (Zlotorynski et al., 2003; Tubbs et al., 2018). Although breakage frequencies and general sequence characteristics have been described for these sites, a common causative sequence has yet to be identified. In most CFSs, several factors associated with the AT-rich sequence likely contribute to the propensity to break under replicative stress. Some mapped CFS loci, such as FRA3B and FRA6E, are coincident with repeat expansions, which could contribute to the increased frequency of fragile site expression at these specific loci under replicative stress. For RFSs, the distamycin-A/BrdU-inducible sites FRA10B/FRA16B map to uninterrupted AT-rich repeat motifs spanning several kilobases (Lukusa and Fryns, 2008). FRA16B has been mapped to a 33 bp AT-rich minisatellite repeat (Yu et al., 1997) as well as a 35 bp repeat (Yamauchi et al., 2000). Differences in repeat motif length or composition between different FRA10B families indicate multiple independent expansion events (Hewett et al., 1998). That the expanded repeats at FRA10B and FRA16B can be of various repeat motifs with various lengths seen among different individuals, supports the likelihood that various AT-rich repeat motifs - when expanded - may become fragile sites. Currently, these AT-rich expansions have not been demonstrated as requirements for fragile site expression. Interestingly, several repeat expansion diseases, such as SCA10, SCA37, FAME1/BAFME, and SCA31, are all caused by pentanucleotide AT-rich repeats that reside within known distamycin rare or aphidicolin CFS regions. At each of these loci, multiple repeat motifs can arise, but only certain motifs are associated with disease (Ishiura and Tsuji, 2020). Additionally, there are also several repeat expansion disease loci in regions not yet associated with fragile sites which could show fragility in patient populations not currently analyzed. We propose that in the large regions associated with CFS, the repeat expansions, although not necessary, could further enhance fragile site expression.

### Ribosomal repeats and fragility

The tandem arrays of rDNA have been observed as fragile sites and other complex macro-structures (Ferraro et al., 1977; Warmerdam and Wolthuis, 2019; Zhou et al., 2021). Fragility induction at rDNA arrays by aphidicolin and actinomycin-D was recently demonstrated (Zhou et al., 2021). The multiple clusters of tandem ribosomal DNA (rDNA) repeat arrays reside in the short arms of five of the 10 human acrocentric chromosomes, 13, 14, 15, 21, and 22 (Boisvert et al., 2007; McStay, 2016). Acrocentrics have the centromere very near the end of the chromosome, have a long q-arm, a centromere (primary constriction), a stalk (secondary constriction), and a satellited arm (Figure 1G). The stalks and satellites are variably sized heterochromatin structures (Orye, 1974; Cheung et al., 1989; Heliot et al., 1997). The stalks contain the genes for 18S, 5.8S, and 28S ribosomal RNA, which occur as tandem copies, with varying lengths. It is the variation in these lengths that is thought to modulate the length of the chromosome, as this is due predominantly to length variations of the stalk (Orye, 1974; Cheung et al., 1989; Heliot et al., 1997). Each acrocentric has short satellited arms containing three bands: p11, p12, and p13. Bands p11 and p13 are composed of the heterochromatic satellite III and β-satellite repeats. Band p12 contains ∼400 copies of the 43-kb rDNA repeat unit tandemly arrayed. Each unit contains the 28S, 5.8S, and 18S rRNAs (45S rRNA) and a non-coding intergenic spacer. The size of the rDNA arrays varies between individuals and decreases with ageing and displays increased length variation in cancers (Stults et al., 2009; Xu et al., 2017; Salim and Gerton, 2019; Valori et al., 2020). The tandem arrays of rDNA are in nucleolar organizer regions (NORs) which are within nucleoli. The exact sequence of the rDNA arrays have long been elusive, but are now able to be known (Hori et al., 2021; Nurk et al., 2022). The chromatin compaction of the rDNA arrays is altered between active and inactive states. In *Xenopus Laevis* the transcriptionally active rDNA arrays are densely compacted in nuclease resistant chromatin (Spadafora et al., 1979; Spadafora and Crippa, 1984; Spadafora and Riccardi, 1985). NORs on metaphase chromosomes present as achromatic gaps known as secondary constrictions of undercondensed rDNA repeats within active NORs (Heliot et al., 1997). The lengths of the rDNA arrays have long been known to contract over aging, especially in the brain (Johnson and Strehler, 1972). The rDNA arrays are particularly unstable in cancers (Stults et al., 2009; Xu et al., 2017; Salim and Gerton, 2019; Valori et al., 2020), and sensitive to DNA damage (van Sluis and McStay, 2019, 2017; Salim et al., 2017). Recent advances on understanding the mechanisms of rDNA fragility have been made, revealing an involvement of transcription across the arrays and R-loop formation (Zhou et al., 2021). The D4Z4 repeat constitutes a family of subtelomeric repeats present on human chromosomes 10q26, 1p12, and the p arm of all five acrocentric chromosomes (Lyle et al., 1995; Stout et al., 1999). Telomeres avoid the nuclear periphery and tend to reside within the internal, euchromatic compartment. Exceptions to this are the telomeric q-arm 4q35 (Tam et al., 2004) and the short p-arms of the acrocentric chromosomes, 13, 14, 15, 21, and 22 (Boisvert et al., 2007; McStay, 2016). Interestingly, each of these harbors a D4Z4 repeat (Lyle et al., 1995; Stout et al., 1999). FSHD patient cells with a mutant contracted D4Z4 repeat tract (typically 11–100 repeats, down to <11 units) still colocalized to the nuclear periphery, arguing that a critical number of D4Z4 repeats is not required for localizing 4q35 (Tam et al., 2004).

### Multi-branched and despiralized chromosomes: Satellites I-III, α-satellite repeats, and ICF syndrome

Human centromeres are composed primarily of repeating ∼171 bp units known as α-satellite DNA repeats (Warburton et al., 1996). Centromeric regions are the primary constrictions of chromosomes and exhibit a high degree of heterogeneity in repeat sequence composition among individuals (Fowler et al., 1987; Altemose et al., 2014; Aldrup-MacDonald et al., 2016). Unlike many fragile sites and repeat expansion diseases, these variations are considered benign. Flanking the centromeres are pericentromeric regions, which are composed of α-satellites and other repetitive elements such as LINES, SINES, and satellites II and III (reviewed in (Plohl et al., 2014). The pericentromeric regions of chromosomes 1, 9, and 16 have large constitutive heterochromatin stretches of repetitive DNAs (*see* Box 1). These regions give rise to the secondary constrictions or stretched heterochromatic sites, that often appear as long over-stretched despiralized regions (Jeanpierre et al., 1993; Guttenbach and Schmid, 1994). These are constitutively seen in patients with ICF syndrome, a rare autosomal recessive disease characterized by immunodeficiency (Fryns et al., 1981; Turleau et al., 1989; Tuck-Muller et al., 2000). Like common and rare fragile sites, these chromosomal regions are prone to breakage, mis-segregation, aneuploidy, and micronuclei formation. Multi-branched inter-chromosomal associations, much like satellite chromosome associations are often observed in ICF chromosomes (*see* Box 1). ICF syndrome is caused predominantly by mutations in *DNMT3b* (the gene encoding the human *de novo* DNA methyltransferase) but also by mutations in the *HELLS*, *CDCA7*, and *ZBTB24* genes, each involved in DNA methylation regulation (reviewed in (Wijmenga et al., 2000). ICF individuals show severe immunodeficiency, abnormal facial features, and cognitive disabilities. All ICF patients assessed to date have hypomethylation of the juxtacentromeric satellite II repeats, leading to the hypothesis that the chromosome fragility and disease symptoms are directly linked to DNA hypomethylation (Maraschio et al., 1988; Jeanpierre et al., 1993). Juxtacentromeric heterochromatin, unlike pericentromeric regions, does not include the centromeric heterochromatin. The cytogenetic observation of despiralized lesions, cytogenetically similar to fragile sites within these specific heterochromatic regions, highlights the importance of methylation in relation to fragility at various loci. This connection is supported by the observation that exposure of non-ICF cells to demethylating agents such as 5-azadeoxycytidine, leads to the induction of the same fragile sites as those endogenously expressed in ICF patient cells (Sutherland et al., 1985b). Furthermore, under replicative stress, such as in tumorigenesis, centromeric DNA rearrangements and mutations are commonly observed, just like at CFS regions. Whether the mechanisms of maintaining chromatin integrity at these various repetitive regions share common pathways has yet to be elucidated. It is notable that other inter-chromosomal associations have been reported by molecular means (Maass et al., 2019; Agelopoulos et al., 2021), however, these have not been reported to be detectable cytogenetically.

### Telomere repeat lesions

The telomeric ends of chromosomes are another site of constitutive, repetitive heterochromatin within the genome. In an attempt to identify internal controls for diagnostic FXS by FRAXA induction, telomeric fragile sites were observed (Steinbach et al., 1982). These folate-sensitive telomere fragile sites occurred more often at 4p, than on other chromosomes (Jenkins et al., 1986a). The cause of this telomeric fragility was not mapped at the sequence level, but their variable expression might be due to chromosome arm specific sub-telomeric sequences (Flint et al., 1997). Telomere repeat tracts (TTAGGG)n are typically bound and protected by the shelterin protein complex, have also been identified as aphidicolin inducible fragile sites (Sfeir et al., 2009; Bosco and de Lange, 2012). The repetitive nature of these long sequences challenges the fidelity of the replication machinery. Deletions of TRF1, a key protein of the shelterin complex, is sufficient to cause telomeric fragile sites similar in appearance to traditional fragile sites induced in replicative stress conditions (Sfeir et al., 2009). This effect can be further exacerbated in aphidicolin or ATR-knock-down replicative stress conditions (Sfeir et al., 2009). The fragile nature of this repetitive sequence is further validated by the presence of fragility at the interstitial telomeric repeat on chromosome 2q14 (Bosco and de Lange, 2012), where two stretches of TTAGGG repeats exist as remnants of telomere-telomere fusions from ancestral ape chromosomes (IJdo et al., 1991). Telomere fragility may be regulated by progerin and dNTP pools (Kychygina et al., 2021). These data argue that the telomere repeat sequence itself is prone to fragility and may share many characteristics with other fragile sites, likely due to its replicative stress response.

### Virally-induced fragile sites at repetitive tracts

Viral integration into the genome has been associated in two ways with fragile sites. First, the integration of foreign DNA, including viruses and plasmids, occurs preferentially at known CFSs (Wilke et al., 1996). This selectivity has previously been harnessed to map the locations of fragile sites (Chen et al., 1976; De Ambrosis et al., 1992; Smith et al., 1992; Wilke et al., 1996; Mishmar et al., 1998). Secondly, several herpes viruses (HSV-1 and HSV-2), papilloma virus (HPV18) (Popescu and DiPaolo, 1989; Zimonjic et al., 1994), cytomegalovirus (Fortunato and Spector, 2003; Siew et al., 2009), and the oncogenic adenoviruses (Ad5 and Ad12) have been reported to induce fragile sites following integration at locations which do not normally express fragile sites (reviewed in (Fortunato and Spector, 2003). Similarly, the integration of foreign DNA into the genome can induce novel fragile sites (Matzner et al., 2003). Virally-induced fragile sites occur without chemical induction, although they can be enhanced following viral integration (Caporossi et al., 1991). The best studied of these virally-induced fragile sites are those induced by adenoviruses. Adenovirus serotype 12 induces fragile sites at four specific genomic locations where viral integration occurs at tandem repeating units. These repeats need to be actively transcribed for fragility to arise (Gargano et al., 1995; Li et al., 1998). One of the earliest studies to observe and map the location of a virally-induced fragile site was through HSV-1 and -2 induced fragile sites at the secondary constrictions of chromosomes 1, 9, and 16, each composed of satellites I-III (Fortunato and Spector, 2003). The HSV infections ultimately lead to random chromosome pulverization/fragmentation (Fortunato and Spector, 2003). It is noteworthy that many of the fragile sites that are claimed to be virally-induced often appear to express fragile sites naturally, but can be induced by exposure to demethylating agents, or in cells from an ICF-affected individual who is genetically deficient in the *de novo* methyltransferase, *DNMT3B* (*see* section 2.3). In this situation, it is difficult to know if cells expressing some of these fragile sites have a history of exposure to these viruses.

## Folate metabolism and fragility

Fragile site expression due to thymidylate stress can be achieved through numerous induction methods that perturb the folate metabolism pathway: *1*) folic acid deficient growth medium; *2*) addition of methotrexate, an inhibitor of dihydrofolate reductase (DHFR); *3*) addition of fluorodeoxyuridine (FUdR), an inhibitor of thymidylate synthase; *4*) excess thymidine, which inhibits the ability of ribonucleotide reductase to convert cytidine diphosphate to deoxycytidine diphosphate, and inhibiting dCTP production (Jacky et al., 1991). Curiously, excess BrdU (a thymidine analog), which also decreases dCTP levels, prohibits FSFS expression (Sutherland et al., 1985b), likely due to its ability to base pair with guanosine in its enol form (Freese, 1959). This pairing allows DNA synthesis to proceed, unlike the excess thymidine treatment that leaves many guanosine molecules unpaired due to dCTP depletion (Sutherland et al., 1985a). Additionally, imbalances in dNTP pools compromise the fidelity of DNA polymerases (Das et al., 1985), increasing mutagenic products *in cellulo* (Mattano et al., 1990; Kunz and Kohalmi, 1991)*,* a pre-disposing factor for fragile site expression*.*

### Folate and DNA methylation

**FIGURE 6:**
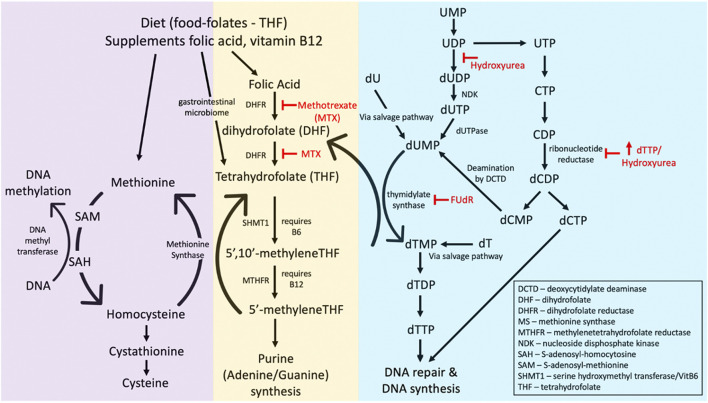
The folate metabolism pathway affecting folate-sensitive fragile site expression. Common fragile site inducers methotrexate (MTX), 5′-fluorodeoyuridine (FUdR), deoxythymidine triphosphate (dTTP), and hydroxyurea are indicated (in red) as to what enzymes they inhibit. Abbreviations of other enzymes or substrates in the pathway are given in the bottom right corner.

The folate pathway is tightly linked with the DNA methylation pathway (Figure 6), suggesting a possible association between folate-sensitive DNA sites and the ICF-linked fragile sites covered in Section 2.3. Folic acid is a cofactor necessary in the methylation of uridine monophosphate (dUMP) to thymidine monophosphate (TMP). Through this conversion of uracil, folate prevents the toxic incorporation of uracil into genomic DNA. The folate metabolism pathway and the various folate stressors are schematized in Figure 6. Experimental evidence suggests that incorporation of uracil into the DNA gives rise to single- and double-stranded breaks, chromosomal breakage, and micronuclei formation (Blount and Ames, 1995; Blount et al., 1997; Duthie and McMillan, 1997; Duthie and Hawdon, 1998). Folic acid also has critical roles in the production of methionine and S-adenosyl methionine (SAM), a methyl donor necessary for many methylation reactions, including the maintenance of DNA methylation (Zingg and Jones, 1997). That four of the eight known human glycosylases exist specifically to remove uracil (UNG, TDG, hSMUG1, MBD4) highlights the toxicity of uracil within the genome (Lindahl and Wood, 1999).

*In vivo* effects of folate upon DNA methylation have been documented in human and animal model studies, where low levels of either dietary or serum folate are significantly correlated with global DNA hypomethylation (Bekdash, 2021). Folic acid supplementation of a low folate diet over a few weeks increased genome DNA methylation (Jacob et al., 1998). Mild folate depletion caused various chromosomal rearrangements in cultured rodent prostate cells, a cell type sensitive to folate deficiency due to its high dependence on SAM for polyamine biosynthesis (Bistulfi et al., 2010). Overall, although it is difficult to observe the *in vivo* effects of folate deprivation, several studies on various cell types suggest genomic instability as a key feature.

### (CGG) repeats and folate depletion

CGG repeats show preferential sensitivity to fragility following folate depletion. While the nature of this sensitivity remains an enigma, one hypothesis focuses on the incorporation of uracil into DNA due to increased dUTP levels. Methotrexate treatment in culture causes a large increase in the dUTP/dTTP ratio, leading to a highly increased incorporation of uracil in DNA (Goulian et al., 1980). This uracil incorporation occurs more frequently in late than in early replicating genes in *S. cerevisiae* (Bryan et al., 2014) and coincidentally, most fragile sites tend to be late replicating (Webb, 1992; Hansen et al., 1993; Subramanian et al., 1996). Therefore, these FSFSs could be stuck in a recurring DNA repair cycle, attempting to excise and replace the uracil base but lacking sufficient levels of the correct dNTP (Reidy, 1987). This cycle is likely exacerbated by cytosine deamination, a naturally occurring process that increases the uracil content at CGG repeats (Feng and Chakraborty, 2017). Alternatively, certain DNA glycosylases function more efficiently at sites of DNA damage when the template contains kinks, bubbles, or gaps that are typical of secondary structures (Hedglin et al., 2015). Therefore, the higher propensity of CGG repeats to form secondary structure could allow uracil DNA glycosylase to more readily recognize misincorporated uracil, thereby setting off or exacerbating a futile DNA repair cycle (Feng and Chakraborty, 2017).

At the time of the earliest predictions of a repeat expansion (*see* Box 2), in 1985–86 (Sutherland et al., 1985b; Nussbaum et al., 1986; Sutherland and Baker, 1986), it was known that perturbation of one nucleotide precursor affected the levels of other nucleotides (Kunkel et al., 1982; Kunz, 1982; Meuth, 1984). Moreover, it was known that nucleotide pool perturbations can lead to altered mutation rates, another phenomenon that is better understood now (Kunz, 1988; Mathews, 2015, 2014, 2006; Mannava et al., 2013). Even damage to the nucleotide precursors themselves alters mutation rates, a phenomenon that may affect repeat instability itself (De Luca et al., 2008; Cilli et al., 2016; Mathews, 2017). However, even today, an appreciation of the precise levels of nucleotides in a cell, their effect upon each another and sub-cellular localization is poorly understood (Leeds et al., 1985; Andersson et al., 1988). This knowledge gap also extends to nucleotide activity-based localization (Mathews and Ji, 1992) and tissue- or development-specific nucleotide pool regulation (Mathews, 2019, 1975; Brachet, 1977). The role of folate in maintaining uracil levels, outlined above, likely plays a role in some of these cellular processes, a connection that will be revealed as researchers seek to better understand the connection between nucleotide levels, repeats, and fragility.

Box 2 **Extended history of FXS**

Based upon the knowledge that FRAXA and other folate-sensitive sites could be induced by perturbing nucleotide pools in the folate pathway (*see* Figure 6), Grant Sutherland’s group hypothesized in 1985 – 6 years prior to molecular proof–that the genetic cause of fragile sites would be an amplified repeat sequence (G. Sutherland, Baker, et al., 1985a; G. R. Sutherland and Baker, 1986). The repeat motif was suggested to be, but not necessarily limited to, amplified alternating polypurine/polypyrimidine sequence, (AG)n•

(CT)n at the fragile site. In 1986, Nussbaum and others further extended this amplified repeat-centric hypothesis to the genetics of FXS (Nussbaum et al., 1986). Specifically, they suggested that carrier females inheriting the amplification would have a level of clinical expression that depended upon the proportion of active X *versus* inactive X chromosomes harboring the repeat amplification (Nussbaum et al., 1986). This suggestion was consistent with the intermediate “premutation” state originally proposed in an effort to explain the puzzling genetic transmission of the disease. The puzzle originated as the cytogenetic fragile site was present in seemingly unaffected males, who would give rise to a definitive mutation only upon transmission to their heterozygous daughters, who themselves were rarely intellectually affected, but went on to have sons with both the fragile site expressed and the disease phenotype with near unity in incidence (Pembrey et al., 1985).

It would be 5 years before landmark back-to-back papers revealed the first evidence that genetic instability was in fact the cause of FRAXA and FXS, demonstrating the increasing size of the disease-causing DNA fragment through transmissions (Oberlé et al., 1991; S. Yu et al., 1991). Both papers suggested the involvement of an expanding repeat tract, and Oberlé specifically suggested the involvement of the CGG tract. These papers were quickly followed by those from Verkerk and others 1991) and Kremer and others 1991) showing a CGG tract was expanding (Kremer et al., 1991; Verkerk et al., 1991). Verkerk identified the novel *FMR1* gene in which the repeat expansion resided. These early papers presented evidence for the mutation mechanism in FXS being an unstable DNA, with somatic instability of the DNA, and proposed the involvement of the GC-rich repeat and unusual DNA structures in the mutation process. Thus, the suspicion of an unstable repeat hypothesized by earlier papers (G. Sutherland, Baker, et al., 1985a; G. R. Sutherland et al., 1986b; Nussbaum et al., 1986) was confirmed in a flurry of papers published within months of each other, revealing that the expansion of the CGG repeat was the cause (Fu et al., 1991; Kremer et al., 1991; Verkerk et al., 1991). The unusual genetics of FXS was subsequently shown to be caused by the size of CGG expansions (Fu et al., 1991; Heitz et al., 1992) as well as the proportion of mutant chromosomes with aberrant methylation being present on the active X of females (Rousseau et al., 1991a). The mode of instability was revealed to be due to somatic repeat instability during early development (Devys et al., 1992). The observed aberrant CpG methylation of the mutant locus (Bell et al., 1991; Oberlé et al., 1991; Vincent et al., 1991) was soon after revealed to be associated with loss of *FMR1* transcription (Pieretti et al., 1991). The identification of the CGG expansion had immediate implications on direct molecular diagnostic methods (Rousseau et al., 1991b; Shapiro, 1991; G. R. Sutherland et al., 1991), improving upon the cytogenetic diagnosis of the previous decade (Veenema et al., 1988; Shapiro, 1991; Shapiro et al., 1991). Thus, a strong sense of biology and genetics can lead to likely hypotheses, yet strong molecular genetics are needed to prove them. *See* also Figures 2, 3.

### Martin-Bell syndrome/FXS

Martin-Bell syndrome, first described in 1943, was the first reported example of X-linked intellectual disability (ID) (Martin and Bell, 1943). The authors noted the unusual transmission by what appeared to be unaffected fathers and mothers. Notably, Julia Bell, a pioneer geneticist and statistician, had previously studied the unusual transmission of both myotonic dystrophy and Huntington’s disease, termed then as “antedating,” now more commonly referred to as anticipation (Bell, 1941). Later, upon examining another multi-generation family with X-linked ID, Lubs identified the first disease-linked fragile site, mapping to Xq27 (Lubs, 1969). This observation eventually led to the name “fragile X syndrome” (FXS). Sutherland revealed in 1977 that fragile site expression occurred in specific culture conditions (Sutherland, 1977), and subsequently several families of X-linked intellectually impaired families were reported to express the same fragile site (Harvey et al., 1977; Turner et al., 1980b, 1980a; Jacobs et al., 1980). The linkage between Martin-Bell syndrome and FXS was definitively made in 1981 when fragile X expression was demonstrated in the same family described by Martin and Bell (Richards et al., 1981). This rapidly lead to harnessing this cytogenetic observation as a diagnostic tool (Webb et al., 1981).

In the decade following 1981’s exciting discoveries, was the race to discover the molecular cause of the FRAXA fragile site and our understanding of FXS and its curious genetics. Based upon the biology of FRAXA induction, perturbation of nucleotide pools, led to the hypotheses that long amplified DNA repeat tracts were the cause of the FRAXA fragile site (Sutherland et al., 1985a; Nussbaum et al., 1986; Sutherland and Baker, 1986; Warren et al., 1987; Hori et al., 1988). Through exceptional and creative molecular and cellular experimentation by multiple groups, in the span of a few months in 1991, a series of papers collectively captured the involvement of an expanding tandem repeat tract with CGG sequence motif, whose expression was affected by aberrant repeat tract methylation, and in females, X-inactivation ratio. The timelines of these discoveries is expanded upon in Box 2, and are detailed further in (Depienne and Mandel, 2021; Gall-Duncan et al., 2022). *See* also Figure 3.

Currently, independent repeat expansion detection methods have confirmed the suspicion that most rare FSFSs are amplified CGG tracts (Garg et al., 2020; Trost et al., 2020). Recent genomic/bioinformatic and epigenetic approaches have colocalized CGG expansions to regions that have previously presented by cytogenetics as fragile sites, although none were validated by cytogenetic FISH mapping (Garg et al., 2020; Trost et al., 2020). That most appear to be CGG repeats does not exclude the possible involvement of other GC-rich motifs. Chromosomal confirmation and association of FSFS with disease phenotypes seems to have revived interest in these repeats.

### Megaloblastic anemia, fragile sites, and folate or B12 deficiencies

Dietary compounds, environmental mutagen exposure, and chemotherapy are strongly correlated with increased FS expression within aphidicolin-treated peripheral lymphocytes (Kao-Shan et al., 1987; Sbrana and Musio, 1995; Musio and Sbrana, 1997; Richards, 2001; Stein et al., 2002; Francés et al., 2016). The list of potential environmental mutagen exposures is extensive and includes cigarette smoke, caffeine, ethanol, lysergic acid diethylamide (LSD), dilantin, pesticides, oil spills, dietary changes, and radiation (therapeutic and atomic bombs). While aphidicolin is still necessary for FS expression in this system, the current knowledge of how such exposures can lead to specific mutation signatures (Poon et al., 2014) may reveal trends related to fragility susceptibility. The increased scientific focus on environmental and dietary exposures may yield additional information on their association with chromosomal fragility.

Folate metabolism depends upon dietary folates (mostly tetrahydrofolate), folic acid as supplements, and vitamins B6 and B12. Humans are not capable of *de novo* production of folate, but the commensal microbiome can support production of this micronutrient. Deficiencies of vitamin B12 and/or folate, due to malnourishment or genetic defects in folate absorption/metabolism, lead to striking chromosomal aberrations in both direct marrow peripheral blood preparations, observations dating back to the 1950s (Cingam et al., 2017; Green and Datta Mitra, 2017). Chromosomal lesions include fragile sites (gaps and breaks), centromere spreading, and chromosome elongation/contraction (Heath, 1966; Jensen and Friis-Moller, 1967; Das et al., 2005, 1986). Numerical (ploidy) was unaltered. Upon proper nourishment or vitamin supplementation, the chromosomal aberrations were rescued, and hence reversible. Moreover, there is extensive knowledge of how certain drugs can lead to megaloblastic anemia where many of the drugs perturb folate, purine, or pyrimidine metabolism, with some overlap with RFSFS-inducers (Stebbins et al., 1973; Stebbins and Bertino, 1976; Hesdorffer and Longo, 2016, 2015; Ben Salem et al., 2016). Aside from the centromere, it is unknown if these chromosomal lesions arise at random or preferred chromosomal locations. The *in vivo* association with folate-deficiencies and the overlap of some drug inducers of chromosomal aberrations in megaloblastic anemia with FSFSs in cultured cells begs the question as to whether there may be molecular similarity to the sequences at the lesions. It is tempting to speculate that tracts of certain expanded repeats may be particularly sensitive fragile site induction upon perturbation of folate metabolism, drawing a direct parallel of disease-associated CGG expanded fragile sites and fragility in megaloblastic anemia. It is notable to some reports of localized mosaic chromosomal rearrangements, where the same rearrangement was observed in multiple metaphases [del(7q), del(3p), del(18p), del(20q)], and in malnourished individuals (Goh, 1981; Chintagumpala et al., 1996; Wollman et al., 1996; Parmentier et al., 2012; Cingam et al., 2017). And in each case the rearrangement was “reversible” upon treatment–indicating that the rearrangement was a folate-sensitive *de novo* event, that did not occur in the presence of folate. A similar link of low blood folate levels and a del(10)(q23), breaking at 10q23, a known folate-sensitive CGG FS FRA10A (Sarafidou et al., 2004), has been reported to be decreased upon vitamin supplementation (Maltby and Higgins, 1987; Ozisik et al., 1994; De Leon-Luis et al., 2005; Morel et al., 2005). It would be of interest to map the locations of the fragile sites in megaloblastic anemias.

## Characteristics of fragile sites

**FIGURE 7:**
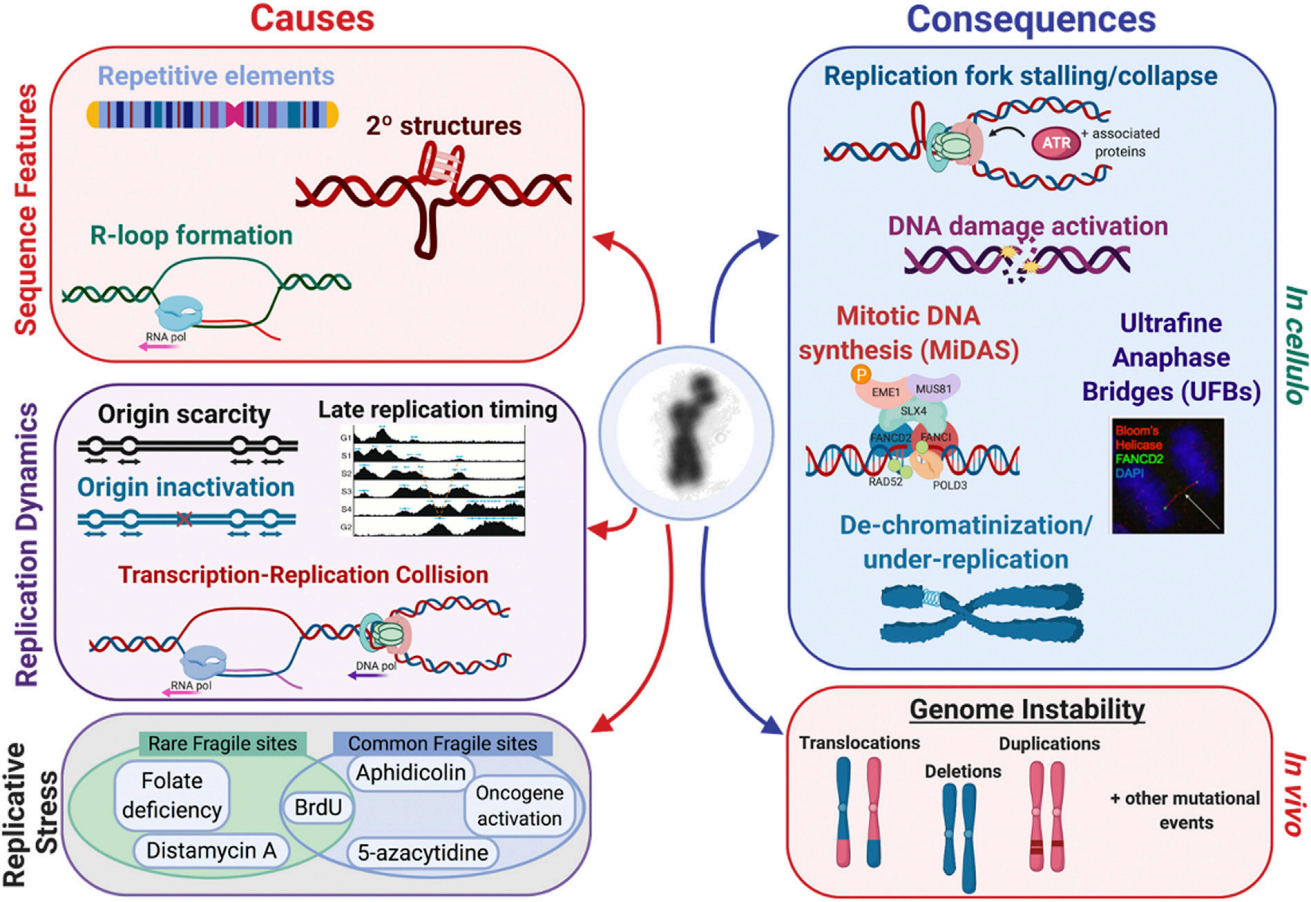
Fragile site expression is caused by several factors, such as *1*) sequence features, *2*) replication dynamics, and *3*) replicative stress conditions. There are several inducers that act on several fragile sites, like FRA3B which can be induced by FUdR and aphidicolin (Kähkönen et al., 1989; Jenkins et al., 1990; Kuwano et al., 1990). There are many consequences both *in cellulo* and *in vivo* that are linked to fragile site expression. *In cellulo*, replication fork stalling and collapse leads to activation of the DNA damage response, mitotic DNA synthesis (MiDAS), ultrafine anaphase bridges (UFBs), and ultimately the appearance of de-chromatinization and under-replication if the site is not repaired in time. This can be observed *in vivo* through mutational events such as copy number variants, translocations, deletions, and duplications.

Common characteristics identified amongst the various types of fragile sites provide critical clues as to why and how fragility occurs at these specific loci throughout the genome. Several of the proposed mechanisms of fragile site formation and resolution are supported by evidence provided by these common characteristics (*see* Figure 7).

### Formation of secondary DNA/RNA structures

All fragile sites have a propensity to form higher order secondary structures more than non-fragile regions of the genome. CFSs, which are typically AT-rich, possess high DNA torsional flexibility (Chen et al., 1985), which influences formation of secondary structures and can perturb DNA replication (Zlotorynski et al., 2003; Dillon et al., 2013). In *S. cerevisiae* AT-rich regions cause fork stalling and breakage (Zhang and Freudenreich, 2007). Work by Burrow and others (2010) shows that only 14 copies of the 33 bp AT-rich minisatellite repeat of FRA16B is enough to cause replication fork stalling, regression, and polymerase skipping *in vitro* (Burrow et al., 2010). Additionally, there is a significant effect on replication due to the orientation and distance of this sequence from the replication origin with electron microscopy revealing spontaneous regression of stalled forks at these sequences (Burrow et al., 2010). While the AT-rich flexible motifs exist within or near deletion breakpoints at fragile sites (Finnis et al., 2005; Burrow et al., 2009), deletion of these motifs within FRA16D (Finnis et al., 2005) or FRA3B (Corbin et al., 2002; Durkin et al., 2008) does not block fragile site expression. This disparity suggests that higher order structures cause by AT-rich motifs at these regions cannot solely explain their fragility. FSFSs, on the other hand, are comprised of expanded CGG repeats that are capable of forming hairpins, slipped strand structures, G-quadruplexes, and *i-*motif structures that can hinder replication fork progression both *in vitro* and *in vivo* (Fry and Loeb, 1994; Kang et al., 1995; Usdin and Woodford, 1995; Samadashwily et al., 1997; Zamiri et al., 2018, 2015). Both rare and common fragile sites form secondary structures, albeit through very different repeat composition, with RFSs having a high predisposition to expand to greater repeat sizes (Schwartz et al., 2006).

### CpG methylation

DNA methylation status has been primarily explored in relation to RFSs, primarily FSFSs, which undergo CpG methylation at the expanded CGG repeats. Generally, folate deficiency decreases methylation levels of the DNA, because without folate, S-adenosylmethionine (the principle methyl donor) is not produced, leading to a reduction of cytosine methylation in the DNA (Giovannucci et al., 1993). All 10 mapped FSFSs are predisposed to aberrant CpG methylation which is also linked with silencing of the associated gene and development of disease phenotype. For some loci, expansions without methylation can lead to different phenotypes all together (*see* Section 4.4). Additionally, the FRAXA, FRAXE, and FRA12A fragile sites have been cytogenetically observed in individuals with unmethylated expanded alleles (Smeets et al., 1995; Perroni et al., 1996; Winnepenninckx et al., 2007), suggesting that methylation is not an absolute requirement for fragile site expression. However, a larger study of high-functioning males with full CGG expansions and considerably reduced aberrant CpG methylation, reveals that fragile site expression does correlate with methylation levels (Hagerman et al., 1994; Rousseau et al., 1994; Lesca et al., 2003). Thus, while DNA methylation is not required for fragile site expression, it can enhance fragility.

It is likely that CFSs are also sensitive to altered methylation status, which could give rise to DNA conformational changes or altered DNA-protein interactions that contribute to fragile site expression (Thys et al., 2015). Interestingly, cytogenetically, fragile sites appear similar to the chromosome constrictions that endogenously arise in cells of individuals with ICF, most of whom are genetically deficient in the *de novo* DNA methyltransferase (*DNMT3B*) (Figure 1). Therefore, methylation likely plays an important role in secondary structure and stability of certain DNA regions, including both FSFSs and at satellite I-III repeat sequences associated with CFS. Perturbation of methylation status at these loci likely increases the propensity for fragile site formation.

The demethylating agents, 5-azacytidine and its analog, 5-deoxyazacytidine, are able to induce CFSs. Currently, five have been found, and are predominantly at methylated heterochromatin regions (Sutherland et al., 1985b). These drugs cause widespread demethylation of DNA through both inhibition of DNMT1 and their incorporation into the genome (Christman, 2002). Additionally, since 5-azacytidine results in hypomethylation of heterochromatic satellite repeat regions, it is likely that these regions are also rich in CpG islands. Another CFS-inducing compound that can incorporate into DNA is bromodeoxyuridine (BrdU), a thymidine analog. There are currently seven CFSs and four RFSs found to be inducible by BrdU (Sutherland et al., 1985b, 1980). Neither 5-azacytidine nor BrdU CFSs have been molecularly mapped to a particular repeat motif; however, these regions are proposed to be low complexity, AT-rich repetitive sequences with a high propensity to form secondary structures (Dillon et al., 2013; Thys et al., 2015).

### Unusual heritability/segregation and karyotypic anomalies

All fragile sites are heritable polymorphic sequence variations (Hecht, 1986), which can be inherited on one or both chromosomes (Sutherland, 1981; Izakovic, 1984; Voiculescu et al., 1991; Martínez et al., 2005) and segregate in families (Sutherland, 1982; Smeets et al., 1985; Romain et al., 1986; Sherman and Sutherland, 1986; Müller et al., 1992; Samadder et al., 1993; Hamel et al., 1994). Fragile sites display unusual patterns of segregation that depend upon the transmitting parent. In a meta-analysis, paternal transmission of the rare autosomal folate-sensitive fragile sites (2q11, 2q13, 6p23, 7p11, 8q22, 9p21, 9q31, 9q32, 10q13, 10q23, 11q13, 11q23, 12q13, 16p12, 19p13, 20p11, and 22q13) significantly deviated from the expected 50% Mendelian inheritance ratio, which is reduced by more than five-fold (Sutherland, 1982; Sherman and Sutherland, 1986; Samadder et al., 1993). However, maternal transmission of these same sites did not significantly deviate from the expected 50% ratio (Samadder et al., 1993). Maternal transmission was also observed for FRA16B (16q22), which is induced by distamycin A/berenil and maps to an expanded AT-rich repeat of approximately 33 bp (Müller et al., 1992). The unusual maternally-biased segregation of the X-linked FRAXA, FRAXE, and FRAXF sites, can in part be explained by maternal CGG expansion bias, ratios of X-inactivation, or a predisposition for CGG contractions in the male germline (Fu et al., 1991; Hamel et al., 1994; Malter et al., 1997). The reduced paternal transmissions of the autosomal fragile sites could be due to maternal genomic imprinting, selection against male gametes carrying the fragile site, or selection against paternally-derived zygotes. We note that many of these transmission reports are sparse, with limited independent confirmation. However, we include these reports here, as it is known that such rare observations can have genetic and clinical impact, as highlighted by the historical situation of FXS.

Karyotypic variations involving mosaic gains or losses of the fragile X chromosome have been observed (Figure 5). Several reports observe these mosaics at higher than expected levels and are likely under-reported owing to the absence of associated cytogenetic studies (Fryns and Van den Berghe, 1988; Santos et al., 2003; Dobkin et al., 2009). Both germline and somatic karyotypic anomalies arise in individuals with CGG-expanded *FMR1* X-chromosomes. These anomalies include mosaic cells from a given individual with 46,FRAXA,Y/47,FRAXA, FRAXA,Y (male FXS-Klinefelter syndrome mosaic with an extra fragile chromosome) or 45,X/46,FRAXA,X (female FXS-Turner syndrome mosaic, where the full-mutation fragile X is lost during somatic cell division) (Banes et al., 2003; Dobkin et al., 2009; Froster-Iskenius et al., 1982, p.; Fryns et al., 1983; Milunsky et al., 1993; Seemanová et al., 1985; Shapiro et al., 1994; Tejada et al., 1994). Non-mosaic instances of such anomalies have also been reported, with cells having only 47,FRAXA, FRAXA,Y; 46,FRAXA,X (Filippi et al., 1988; Kupke et al., 1991), or 47,FRAXA,X,X (Fuster et al., 1988; Tejada et al., 1994; Dobkin et al., 2009). These cases can arise *via* either maternal or paternal X-chromosome non-disjunction of the CGG-expanded fragile X chromosome (Santos et al., 2003; Dobkin et al., 2009). Mosaicism occurs when the non-disjunction arises post-zygotically, whereas non-disjunction during meiosis will give rise to homogeneous cell populations. Age-dependent increases of aneuploidy involving the expanded X also occur in most *FMR1* CGG expansion carriers, where the mutant X-chromosome is either lost or retained in an ongoing manner (Nielsen, 1986). FRAXA chromosome aneuploidy is observed in both young and older individuals suggesting that the fragile expanded X chromosome is prone to missegregation (loss or gains), possibly through aberrant packaging, DNA breakage, and/or arrested replication (Kerem et al., 1988; Dobkin et al., 2009; Yudkin et al., 2014). Mosaicism for the ploidy loss or gain of the FRAXA chromosome might suggest meiotic and mitotic predisposed non-disjunction of the mutant chromosome (Milunsky et al., 1993). Such cases can pose diagnostic and counselling challenges (Pandelache et al., 2021). Like mosaics, chromosome number anomalies in cells expressing fragile sites may also be underestimated and overlooked, as chromosome counting has been historically poorly appreciated (Martin, 2004).

Karyotypic variations can also arise with other FSFSs. For example, the FRA1E (1p11) and FRA1D (1p22) fragile sites have been associated with the presentation of monosomy, trisomy, and chromosome rearrangements and multiple congenital anomalies (Neu et al., 1988). In this case mosaicism was evident in multiple tissues including 45,XY,-1/46,XY/47,XY,+1 mosaicism in lymphocytic culture, a 45,XY,-1/46,XY mosaicism in skin fibroblasts, and fra(1p) sites in 2% of the metaphases from lymphocyte, fibroblast, and bone marrow cultures. Given the lack of appreciation for chromosome counting and cytogenetics in an increasingly focused “-omics” world, it is highly likely that other instances of unusual heritability, segregation, and karyotypic anomalies associated with fragile sites remain to be uncovered.

### Disease-association of fragile sites, chromosomal deletions/rearrangements, penetrance, and diversity

The overwhelming association of fragile sites with multiple diseases has fueled their molecular characterization. In particular, CFSs are frequently sites of CNVs and chromosomal rearrangements–deletions or translocations commonly seen in many cancers (Popescu, 1994; Mimori et al., 1999; Krummel et al., 2000; Mangelsdorf et al., 2000; Arlt et al., 2006, 2002; Burrow et al., 2009; Bignell et al., 2010). Many fragile sites also overlap with tumor suppressor genes (Iliopoulos et al., 2006), with rearrangements possibly driving oncogenesis and affect genes that are likely to further accelerate genomic instability (reviewed in (Karras et al., 2016). In addition, oncogenic activation often, due to unchecked cellular growth, causes dNTP imbalances, promoting instability at CFS regions (Bester et al., 2011). Fragile sites are also frequent integration sites of oncogenic viruses (*see* section 2.5), which have been used to facilitate their precise mapping (Smith et al., 1992; Wilke et al., 1996; Mishmar et al., 1998). Finally, fragile site regions are strongly correlated with chromosomal rearrangements that have contributed to the development of the vertebrate lineage, suggesting a link between fragile sites and genome reorganization through evolution (Miró et al., 1987; Ruiz-Herrera et al., 2006, 2005, 2002). These factors suggest a strong connection between fragile sites and both advantageous and deleterious chromosomal processes.

Fragile sites are associated with a number of neurological, neuropsychiatric disorders, and neurodevelopmental diseases such as autosomal recessive juvenile parkinsonism (FRA6E) (Denison et al., 2003), idiopathic autism (FRA13A) (Savelyeva et al., 2006), and schizophrenia (Demirhan et al., 2006). In particular, 28 CFSs contain genes associated with schizophrenia (reviewed in (Smith et al., 2010). There are also claims of fragility linked to bipolar disease, schizophrenia, and Rett syndrome (Archidiacono et al., 1985; Gillberg et al., 1985; Simonic et al., 1997; Fischer, 1998; Demirhan et al., 2009, 2006; Smith et al., 2010; Kharrat et al., 2017). However, despite the historical connection between fragile sites and disease, the reproducibility or genetic mapping of these types of sites has not been sufficiently followed-up.

**FIGURE 8:**
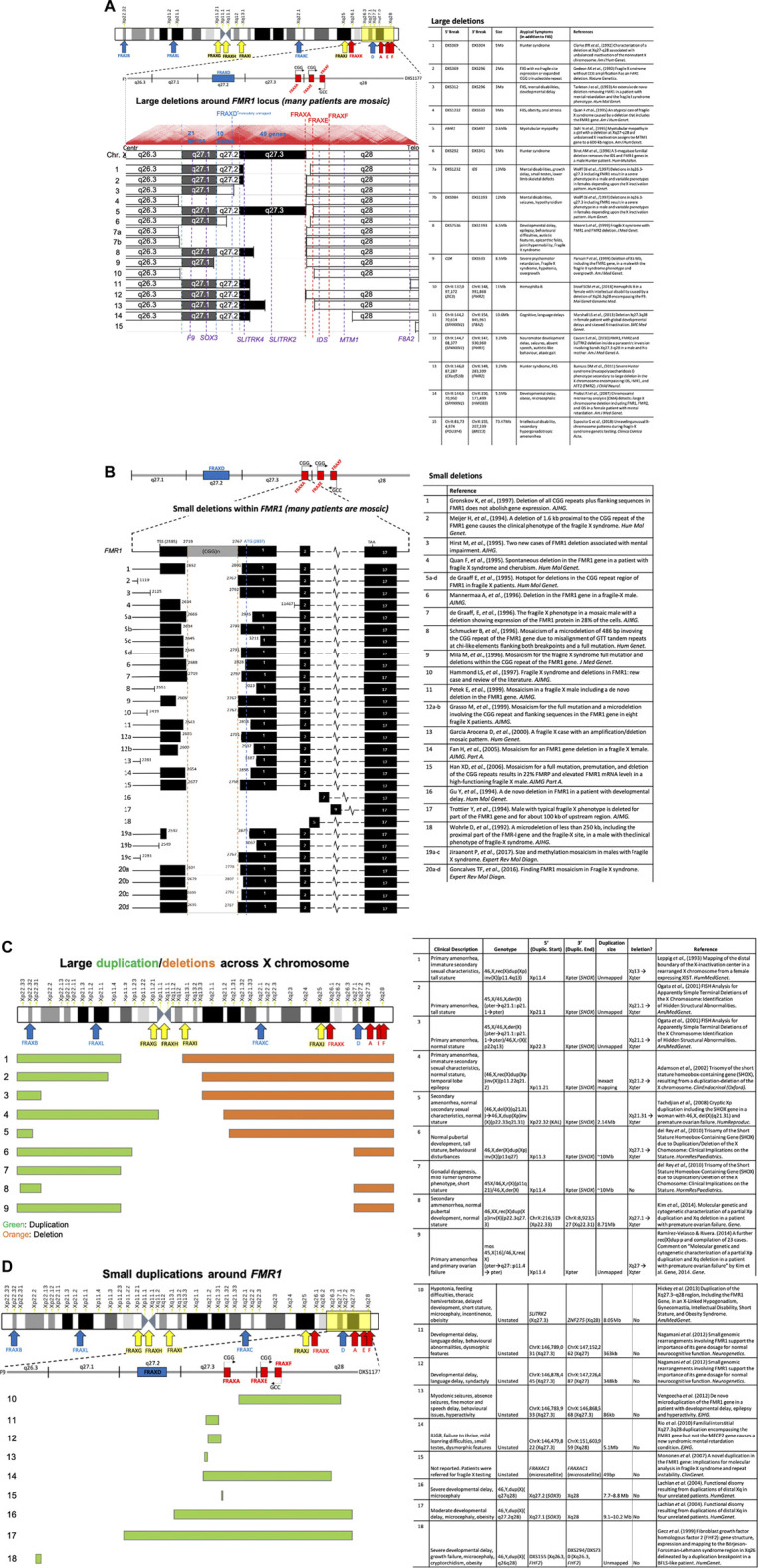
(Continued). Numerous deletions and duplications around the *FMR1* locus. **(A)** The literature reports numerous large deletions on the X chromosome in the region surrounding the *FMR1* locus, and additionally **(B)** many small deletions that occur within the *FMR1* locus itself. Large **(C)** and small **(D)** duplications (including transversions and inversions) are reported to occur around the *FMR1* fragile X locus, with many of the large duplications also occurring in tandem with large deletions (*see* related citations).

Genomic instability at RFSs presents predominantly as expansions of the repeat motif. However, deletions of the FRAXA and FRAXE region do occur (reviewed in (Hammond et al., 1997; Nichol Edamura and Pearson, 2005; Coffee et al., 2008; Mondal et al., 2012)) and have been covered extensively for FRAXA (http://www.hgmd.cf.ac.uk/ac/gene.php?gene=FMR1). Most of the *FMR1* deletions/rearrangements are covered in Figures 8A–D (*see* citations therein). Breakpoints that frequently occur at RFSs, particularly under replicative stress, tend to map to regions surrounding the expanded repeat motif, such as at FRAXA (Warren et al., 1987; Oberlé et al., 1991; Dobkin et al., 2009; Verdyck et al., 2015) and FRA11B (Michaelis et al., 1998; Tunnacliffe et al., 1999). Translocations, deletions, and rearrangements at the fragile X chromosome as well as chromosome 3 were induced under replicative stress using aphidicolin or FUdR, respectively, in somatic cell hybrids (Glover and Stein, 1988). CGG expansion-associated chromosomal deletions can arise somatically and are present at barely detectable mosaic levels, suggesting that the true extent of these deletions may be underappreciated (Jiraanont et al., 2017).

Atypical symptoms also arise with mosaic deletions of *FMR1* and contiguous genes, *FMR1* duplications, and chromosome rearrangements. These rearrangements can be relatively small or large, often bridging fragile site to fragile site. Depending upon the region duplicated or deleted additional symptoms can include hemophilia, Hunter syndrome, myotubular myopathy, overgrowth, macrocephaly, seizures, and others (Figures 8A–D) (Coffee et al., 2008). While FRAXA/FMR1 is heavily studied, numerous instances of chromosomal instability with common and rare fragile sites supports this as a common attribute of FS.

Evidence that the FRAXA site is truly fragile and prone to DNA breakage arises from the many patients that have incurred loss of *FMR1* function through deletions of the (CGG)n tract and part of, or all of the *FMR1* gene, and often contiguous genes (Figures 8A–D). Cytogenetically, FRAXA can manifest as a truncated X chromosome with loss of the distal long arm band, Xq28 (Fitchett and Seabright, 1984; Verdyck et al., 2015). Fragile sites are mutation and epimutation hotspots. Specifically, the (CGG)n-expanded *FMR1* gene incurs ongoing somatic expansions of the (CGG)-tract (Lokanga et al., 2013), variations of CpG methylation, microdeletions, duplications, and point mutations proximal to or encompassing the *FMR1* (CGG)n repeat, intra- and inter-chromosomal rearrangements, as well as germline and somatic aneuploidy (gains and losses of the whole mutant X-chromosome). These various mutations and epimutations, which can arise somatically, can lead to the broad spectrum of phenotypes associated with *FMR1* and its proximal genes (Figures 8A–D). Thus, FSFSs are genetically unstable loci, where the instability can have disease implications.

All of the 10 mapped CGG FSFSs have been linked in some manner to ID or autism spectrum disorders (ASDs) (previously reviewed in (Debacker and Kooy, 2007) (Table 2). Additionally many unmapped fragile sites are proposed to be associated with neurological and neuropsychiatric disorders, including schizophrenia (Debacker and Kooy, 2007). Many of these neurological disorders are complex, polygenic conditions that are heavily influenced by environmental and genetic components (reviewed in (Miles, 2011; Kerner, 2014); therefore, the effect of chromosome fragility on particular genes could cascade to other genes (Feng and Chakraborty, 2017). It is interesting that many of the mapped CGG-repeat expanded FSFSs are from genes that are highly expressed in the brain (*AFF3*, *ZNF713*, *FAM10AC1*, *FMR1*, *FMR2*) which are likely to have many downstream interactions that can affect global gene or protein expression contributing to disease pathogenesis (Feng and Chakraborty, 2017). Molecular mapping of additional fragile site sequences will likely unveil some of the same complexities of disease etiology for their associated diseases.

Jacobsen syndrome occurs due to deletions in the distal end of the q arm of chromosome 11, that is associated with the FRA11B fragile site (Jacobsen et al., 1973; Schinzel, 1977). The clinical presentation is highly variable and can include malformations of the heart, kidney, gastrointestinal tract, genitalia, and central nervous system; cognitive impairment, and skeletal, ocular, hearing, immunological, and hormonal problems. The varying size and locations of the deletions likely account for the variable clinical presentation (Jacobsen et al., 1973; Tootleman et al., 2019). The molecular basis of the deletions is the fragile site FRA11B (CGG)n repeat expansion, which upon transmission can result in breakpoints of the chromosome (Voullaire et al., 1987; Jones et al., 1995). These breakpoints frequently occur within the vicinity of RFS but can also occur up to 10 Mb away from the (CGG)n repeat (Michaelis et al., 1998; Tunnacliffe et al., 1999). This fragile site was the first established as causing *in vivo* breakage and disease manifestation, demonstrating the clinical importance of fragile sites. This connection between *in vivo* breakage and disease is further supported by evidence of FRAXA chromosomal breakage and rearrangement in early embryos containing the repeat expansion (Verdyck et al., 2015). Additionally, expression of the FRA10B fragile site in mothers was correlated with 10qter deletions originating from the FRA10B locus that were identified through non-invasive prenatal screening. Furthermore, the FRA18C CFS was discovered in the parent of an offspring with a chromosomal deletion truncation originating from this site (Debacker et al., 2007). Taken together these data strongly support that fragility is associated with *in vivo* chromosomal breakage and disease manifestation. Some evidence suggests that telomere healing can arise at broken fragile sites, leading to interstitial telomeric sequences (Bosco and de Lange, 2012; Bouffler et al., 1993; Boutouil et al., 1996; Glousker and Lingner, 2021; Gozaly-Chianea et al., 2022, p.; Musio and Mariani, 1999; Petit, 1997; Villa et al., 1997). While there is often proximity of a fragile site to these interstitial telomeric repeats, interstitial telomeric repeats do not necessarily cause fragility (IJdo et al., 1992).

Most rare FSFSs diseases show partial penetrance of clinical symptoms while still expressing the fragile site, a connection that depends upon the presence of a CGG expansion. With the exception of FRAXA/FXS, analysis of penetrance of the other CGG fragile sites has not been possible, as many sites have been observed in too few families to account for either age effects, expansion size, or degrees of aberrant CpG methylation (Debacker and Kooy, 2007). Delayed onset or incomplete penetrance is typical of diseases that display genetic anticipation (earlier manifestation or greater severity through family generations). The FRA16A (CGG)n repeat expansion, initially reported as a benign variation when heterozygous (Nancarrow et al., 1994), is the causative mutation of Baratela-Scott syndrome when found in the homozygous state (LaCroix et al., 2019). Other FSFSs may in fact be found to cause disease when in the inherited in the homozygous state but have yet to be identified due to lack of appropriate patient or patient-derived samples.

Chromosomal fragility has not been observed in other known repeat expansion disease loci outside of (CGG)n expansions. For example, the expanded (CTG)n repeats associated with myotonic dystrophy (DM1) or Huntington’s disease (HD) do not express fragile sites in a variety of induction methods (Beverstock et al., 1985; Jalal et al., 1993; Wenger et al., 1996; Barbé and Finkbeiner, 2022). The DM1 studies used multiple patient-derived cells with long *DMPK* CTG/CAG repeat expansions, and multiple known fragile site induction conditions, including folate-deficient media, high thymidine media, and FUdR (three folate-sensitive rare fragile site conditions), BrdU (rare and common fragile sites), aphidicolin (common fragile sites), and 5-azacytidine (common fragile sites). The HD studies also used multiple HD lines and folate-deficiency, FUdR, and BrdU. Thus, the expansion of any repeat cannot automatically be assumed to lead to fragility. While other chemicals known to induce fragile sites, like the AT-specific Hoechst 33258 and netropsin could be tried, other chemicals, not previously assessed for fragile site induction could also be tried. For example actinomycin D, which has been shown to have loose binding preference to CAG/CTG repeats (Jacky and Dill, 1983) may be considered. Altered protein regulation could be considered. For example, in the presence of an ATM protein inhibitor, the expanded GAA repeat tract at the *FRDA* locus associated with Friedreich’s ataxia, exhibits enhancements in a kind of fragility as assessed through rearrangements and chromosome abnormalities (Kumari et al., 2015). While the association between repeat expansions and fragility has not been a universal association, it is entirely plausible that the unique conditions or agents necessary to induce fragility across different repeat sequences have not yet been elucidated.

### Tissue-/cell-type specific fragile site expression

Fragile site expression has only been demonstrated in a limited number of tissues. Expression of fragile sites is specific to certain cell types, suggesting that epigenetic or other trans-factors could be contributing to the sensitivity of the site to replication stress. For example, although fibroblast cells of fragile X patients can be treated to induce the FRAXA site (Tommerup et al., 1981), the frequency of expression is often not comparable to that seen in the lymphocytes or lymphoblasts of the same patient (Mattei et al., 1981; Schmidt and Passarge, 1986). Differential fragile site expression has additionally been observed between chorionic villus (placental tissue), fetal blood, and amniocytes *via* prenatal fragile X screening (Jenkins et al., 1986b; McKinley et al., 1988). FRAXA expression between a broad range of tissues has yet to be assessed. The specificity of fragile site expression has also been documented for certain CFSs, revealing CFS loci differences across several cell types (Kuwano et al., 1990; Le Tallec et al., 2013, 2011). Letessier and others (2011) demonstrated that cell type specificity (lymphoblastoid *versus* fibroblasts) was linked to the density of replication origins surrounding a particular fragile site region (Letessier et al., 2011). The FRA3B site, the most common CFS site in lymphocytes, has a paucity of replication origins within the core of the region (Palakodeti et al., 2010), yet this disparity does not exist in fibroblasts, where the density of initiation events is comparable to that of the rest of the genome (Letessier et al., 2011). In line with this model, fibroblasts, but not lymphocytes, lack origin sites at the core regions of FRA1L and FRA3L, which are highly expressed in fibroblasts but not lymphocytes (Le Tallec et al., 2011). While cell type specificity has been suggested to be influenced by transcription levels of the particular locus (Helmrich et al., 2011), a later study did not find a correlation with transcription levels, instead suggesting that chromatin architecture and organization play a key role in cell type specificity (Le Tallec et al., 2013). Overall, these findings suggests that although sequence composition is a contributing factor to fragility, there are other undefined aspects influencing the propensity of these sites to experience chromosomal instability. Elucidating these contributing factors could present novel approaches to targeting genomic instability at these problematic loci.

### Micronuclei formation

The increased formation of micronuclei under fragile site inducing conditions has been observed for both rare and common fragile sites (Chan et al., 2009; Bjerregaard et al., 2018). The expression of micronuclei is a proxy for genomic instability, as these events form only after faulty chromosomal segregation in anaphase leads to either an entire chromosome or a chromosome fragment becoming dissociated from the remaining nuclear content (Fenech, 2007). In the absence of drug treatments or external stressors, increased micronuclei appear in cell culture for many neurodegenerative diseases, such as Huntington’s disease (Sathasivam et al., 2001), Alzheimer’s disease (Migliore et al., 1997), ataxia telangiectasia (Rosin and Ochs, 1986), and both Werner and Cockayne syndromes (Weirich-Schwaiger et al., 1994) (reviewed in (Migliore et al., 2011). Micronuclei formation, gene amplification, and chromosome damage (such as double-stranded breaks) appear in conditions of folate deficiency (Jacky et al., 1983; Duncan, 1986; Blount et al., 1997; Melnyk et al., 1999; Fenech, 2001; Fenech and Crott, 2002; Beetstra et al., 2005, p. 200). In conditions of folate deficiency, FXS cells show increased mis-segregation of the FRAXA allele, with a higher prevalence in micronuclei and at anaphase bridges (Bjerregaard et al., 2018). This finding expanded initial reports of increased levels of micronuclei in FRAXA carriers compared to controls (Jacky et al., 1983; Duncan, 1986). Taken together these *in cellulo* reports support a close connection between fragility and micronuclei formation.

The *in cellulo* effect of elevated micronuclei with folate deprivation translates to mouse models and humans. Mice treated with methotrexate, an inhibitor of dihydrofolate reductase (DHFR), exhibit increased micronuclei and chromosomal aberrations in a dose-dependent manner (Kasahara et al., 1992). Additionally, there is a significant correlation between increased micronuclei and folate deficiency in the leukocytes, reticulocytes and erythrocytes of human subjects; supplementation with folate significantly reduced the frequency of micronuclei (Everson et al., 1988; Blount and Ames, 1995; Blount et al., 1997). Recent advances in understanding the biology of micronuclei, including their involvement in DNA damage, aneuploidy, DNA repair and segregation, can be harnessed to further understand the association of micronuclei with fragile sites.

### Sister chromatid exchange and ultrafine anaphase bridges

Sister chromatid exchange (SCE) is a natural phenomenon that occurs following DNA replication and causes recombination of genetic material between chromatids, typically in an error-free manner. Although SCE occurs naturally, an increase in frequency is indicative of genotoxic stress and instability. Advances in understanding the formation and resolution of SCEs, the factors involved, their involvement in genome instability, and under-replicated regions (as observed at FRAXA (Kerem et al., 1988)), all will improve our understanding of the association of SCEs with fragile sites (Baxter, 2015; Broderick and Niedzwiedz, 2015; Uchiyama and Fukui, 2015). CFSs are sites of preferential SCE, regardless of whether a visible gap exists concurrently with the SCE (Glover and Stein, 1987; Schmid et al., 1987; Feichtinger and Schmid, 1989; Hirsch, 1991; Lukusa et al., 1991; Tsuji et al., 1991).

The formation of ultrafine anaphase bridges and the presence of MiDAS (mitotic replicative-stress DNA synthesis, *see* section 5.4) occurring at CFSs has led to great excitement concerning the processing and resolution of SCEs at these sites (Chan et al., 2009; Naim et al., 2013; Ying et al., 2013; Minocherhomji and Hickson, 2014; Bhowmick et al., 2016). Under aphidicolin-induced replicative stress, sister chromatid bridging leads to inefficient resolution and genotoxicity (Chan et al., 2009). These stressed cells have a higher incidence of ultrafine anaphase bridges, indicative of unresolved sister chromatids during anaphase separation (Chan et al., 2007). The FRAXA locus has an increased propensity for DNA anaphase bridges and lagging chromosomes in folate stress conditions (Bjerregaard et al., 2018). These anaphase bridges associate with the FRAXA allele and differ from CFS-associated bridges in that the majority are RPA protein positive and PICH protein negative. FANCD2 also does not colocalize to these bridges, suggesting that FSFSs are processed differently than CFSs (Bjerregaard et al., 2018). This response is outlined in the mechanism of DNA repair section below (section 5.4). Increased sister telomere associations in conditions inducing telomere fragility, suggests a similar pathway at these fragile sites (Sfeir et al., 2009).

The relation between RFSs and SCEs is less clearly delineated, although most studies support an increase in SCEs at RFSs. Carriers of the distamycin A-inducible sites have elevated SCEs, with FRA16B observed in both induced and uninduced conditions (Schmid et al., 1987; Lukusa et al., 1991; Seki et al., 1992), whereas FRA16E is only observed in induced conditions (Tsuji et al., 1991). A problematic aspect in studying SCEs and rare FSFSs is that the BrdU treatment–necessary for SCE visualization–counteracts the toxic effects of folate deprivation by base-pairing with guanine (Freese, 1959). This pairing bypasses the DNA synthesis block normally observed in folate-deprived conditions, where dTTP and dCTP levels are diminished. Reports of a global increase in SCE events in folate-deficient conditions in FXS patient and control cells (Branda et al., 1984), have been countered by others arguing that SCEs are only increased locally at the Xq27 *FRAXA* expanded locus (Wenger et al., 1987; Tommerup, 1989, p. 1989) and global SCEs are the same between these cells. In yeast, thymidylate depletion leads to unequal SCEs and other forms of intrachromosomal rearrangements (Kunz et al., 1986). Further investigation of the relationship between RFSs and SCEs is required to better understand the connection between these two important molecular processes.

The high coincidence of SCEs and fragile sites likely occurs because fragile sites are usually late-replicating, and likely under-replicated, making them susceptible to initiation of homologous recombination to replicate the remaining DNA. If replication is not completed, there will be colocalization of a fragile site with an SCE site. Alternatively, a fragile site could still occur if replication has taken place, but it was too late for proper chromatin condensation. Considering that both rare and common fragile sites are prone to deletions, expansions and rearrangements, the process of unequal or error-prone exchange at SCEs may also contribute to instability.

### Fragile sites in (non-human) animals

Fragile sites have been observed in many non-human species, using induction methods typical of rare or common fragile sites. Aphidicolin-induced fragile sites have been observed in many animals and different cell types. These include, but are not limited to, peripheral lymphocytes from mouse (Rozier et al., 2004), cat (Stone et al., 1993; Stone and Stephens, 1993), and dog (Stone et al., 1991), fibroblasts from mouse (Sanz et al., 1986), Persian vole (Djalali et al., 1985), Chinese hamster (Coquelle et al., 1998), racoon, dogs (Wurster-Hill et al., 1988), and splenocytes from mouse (Krummel et al., 2002) and rat (Robinson and Elder, 1987). Fragile sites are induced by folate deficiency, either through FUdR induction or growth in folate deficient media. These FSFS have been observed in lymphocytes from mouse (Elder and Robinson, 1989), rat (Elder and Robinson, 1989), river buffalo (Pires et al., 1998), Indian mole rat (Tewari et al., 1987), Persian vole (Djalali et al., 1985), goats (Lopez Corrales and Arruga, 1996), cattle (Uchida et al., 1986), and domestic pig (Yang and Long, 1993). Drugs such as trimethoprim, methotrexate, 5-azacytidine, 5-aza-2′deoxycytidine, amethopterin, and BrdU have been used to induce fragile site in non-human animals as well, including in cats (Stone et al., 1993), dogs (Stone et al., 1991), Persian vole (Djalali et al., 1985), gorilla (Schmid et al., 1985), chimpanzee (Schmid et al., 1985), goats (Lopez Corrales and Arruga, 1996), river buffalo (Pires et al., 1998), Chinese hamster (Coquelle et al., 1998), and rabbit (Poulsen and Rønne, 1991). Furthermore, spontaneous (un-induced) fragile sites have been observed in cells from horse and pigs (Riggs and Rønne, 2009).

Evidence suggests that fragile sites and their associated genes are evolutionarily conserved, supporting a functional role, possibly in genome packaging (Berthelot et al., 2015). Many of the human disease-associated genes are evolutionarily conserved and many retain a repeat tract. For example, *FMR1*, for which an expanded CGG tract is the cause of FRAXA, is evolutionarily conserved. Moreover, the CGG repeat is also conserved, but is typically shorter in non-human species. An observation of a naturally-occurring CGG-expanded *Fmr1* gene in a non-human species has not been reported but could theoretically exist. A repeat expansion in the dog *Nhlrc1* gene, the cause of its Lafora disease (Lohi et al., 2005), does not appear to be present in humans, albeit non-repeat mutations in the same gene cause the same Lafora disease in humans (Chan et al., 2003). Human fragile sites have also been conserved within animal species, such as FRA16D, a common AT-rich repeat fragile site mapped to the gene *WWOX* (Lee et al., 2021). In the mouse, the *Wwox* gene and fragile site are highly conserved in the mouse genome, appearing as mouse fragile site Fra8E1 (Krummel et al., 2002). Similarly, a CFS induced by 5-azadeoxycytidine on human chromosome 1q42 was also induced on the homologous locus in chimpanzee and gorilla, indicating that it is also conserved (Schmid et al., 1985). The folate-sensitive FRAXA fragile site at Xq27 was observed in human-hamster and human-mouse hybrid cells, in which a human Xq24-qter from a male fragile X patient was transferred to rodent cells (Nussbaum et al., 1983, p. 983; Warren and Davidson, 1984; Warren et al., 1987). These hybrids were used to clone the FRAXA CGG repeat (Warren and Davidson, 1984; Warren et al., 1987).

## Proposed mechanisms for fragile site formation and processing

The molecular mechanism of fragile site expression remains to be elucidated. Well-established link between fragile sites and cancer etiology has facilitated headway in the field of CFSs, unravelling many mechanistic aspects of their cause and the processing of DNA at these unique sites. In contrast, the field of RFSs lags behind CFS studies and could benefit from ideas gleaned from CFSs to renew progress and discovery. Several theories exist, supported by ample evidence, for the mechanisms of fragile site formation and involve *1*) replication timing and origin paucity, *2*) chromatin compaction, *3*) replication-transcription collisions, and *4*) DNA damage and repair machinery. These pathways are not mutually exclusive, and any combination could cause specific fragile sites, but not necessarily all fragile sites. Some of these causative factors and consequences are briefly summarized in Figure 7 and briefly discussed in the following sections.

### Issues with replication timing and origin paucity

Many common and rare fragile site loci are late replicating regions and often lack nearby replication origins (Le Beau et al., 1998; Wang et al., 1999; Handt et al., 2000; Hellman et al., 2000; Palakodeti et al., 2004; Pelliccia et al., 2008). However, these characteristics are not universal, as some fragile sites are in mid-replicating regions (Handt et al., 2000; El Achkar et al., 2005) and others at early replicating regions (Barlow et al., 2013). FRA3B and FRA16D, the most active CFSs in lymphoblastoid cells, are late replicating with decreased sites of replicative origin within their core regions. As such, more distant replication forks are required to traverse longer distances to eventually complete replication in these regions (Letessier et al., 2011). The same situation applies for FRA6E (Palumbo et al., 2010) and FRA7H, which have allelic asynchrony in replication (Hellman et al., 2000). These regions become particularly vulnerable when stressed with aphidicolin, since the resulting reduction of fork speed has a greater effect on longer-travelling than on shorter-travelling forks (Letessier et al., 2011). In fibroblast cell lines, the FRA3B site does not lack replication origins at this locus, hence the FRA3B fragile site is not expressed in these cells (Letessier et al., 2011). This disparity between lymphoblasts and fibroblasts provides a potential explanation as to the origin of cell-type specificity observed for most fragile sites.

The folate sensitive FRAXA (Xq27.3) and FRAXE (Xq28) regions are both in very late replicating regions in genomes that do not contain the fragile site-causing (CGG)n repeat expansion (Subramanian et al., 1996; Hansen et al., 1997). A CGG expansion at FRAXA obstructs firing from an adjacent replication origin frequently utilized by the wild-type allele. Thus, replication stress at this locus is generated from the need to rely upon more distal origins for replication (Yudkin et al., 2014). The presence of an expansion and additional thymidylate stress delays replication into G2 phase, yielding a large under-replicated region of 1 Mb for FRAXA and 300 kb for FRAXE (Subramanian et al., 1996; Hansen et al., 1997). For these FSFS, the expansion plays a critical role in influencing replication timing and related stress.

Understanding the factors at play during replication will provide clues to the association between fragile sites and replication. The origin replication complex (ORC), which is responsible for directing DNA replication throughout the genome, is assembled at specific loci through an unknown mechanism (reviewed in (Fragkos et al., 2015). Mapping of these complexes using the constituent ORC2 protein in ChIP-seq experiments demonstrated a strong association between regions of CFSs and ORC2 paucity, with 73% of all CFSs upholding this relationship (Miotto et al., 2016). Increased ORC2 correlates with regions of active chromatin, demarcated by higher levels of active transcription and histone marks (Miotto et al., 2016). What determines whether an ORC will fire is a topic of much debate but is predominantly believed to be a stochastic event influenced by factors such as chromatin architecture. A higher density of ORCs likely indicates an early replicating region (Rhind, 2006; Bechhoefer and Rhind, 2012; Gindin et al., 2014; Das et al., 2015), such that the aforementioned ORC2 paucity would be in line with general late replication of fragile sites. Determining the unique characteristics of the chromatin architecture within fragile sites could provide valuable insight into what elements and factors contribute to late replication initiation.

### Chromatin compaction variations

What does the apparent gap, constriction, or break of a fragile site represent at the chromatin level? Some data suggest an uncompacted nucleosome-free DNA (Hsu and Wang, 2002), but can also represent a true physical break within the DNA, or an as-of-yet defined epigenetic factor could be influencing these problematic regions.

Generally, CFSs are hypoacetylated compared to the rest of the genome, indicating that they exist in a compact chromatin form (Koch et al., 2007; Savelyeva and Brueckner, 2014). To probe this nuclear chromatin compaction, a widely utilized endo-exonuclease named micrococcal nuclease (MNase) is employed. MNase preferentially cleaves linker DNA between nucleosomes (Rivera and Ren, 2013; Tsompana and Buck, 2014). FRA3B is more resistant to MNase treatment when compared to non-fragile sequences at or nearby the locus, and demethylating agents trichostatin A or 5-azadeoxycytidine cause a reduction in chromosome breakage at this site (Jiang et al., 2009). Early evidence from the characterization of repetitive satellite DNA sequences from various species demonstrated that these regions are MNase resistant (Bostock et al., 1976; Bowen, 1981).

The FSFS FRAXA displays similar characteristics: *in cellulo,* the FRAXA locus exists as an inaccessible region, resistant to restriction enzyme digestion when compared to the unexpanded allele (Luo et al., 1993; Eberhart and Warren, 1996). This resistance is likely due to the array of repressive histone post-translational modifications that are typically associated with expanded (CGG)n repeats and the *in cellulo* heterochromatin-like state (Coffee et al., 2002, 1999). Fragile site expression is blocked with sodium butyrate and acetyl-carnitine, drugs which inhibits histone deacetylation, encouraging the accumulation of acetylated open chromatin (Pomponi and Neri, 1994). Curiously, *in vitro*, these (CGG)n expanded repeats strongly exclude nucleosome assembly, which is further exacerbated by CpG methylation (Godde et al., 1996; Wang et al., 1996; Wang and Griffith, 1996). Given the challenges in assessing fragile sites at expanded repeats, it is possible that other aberrantly bound DNA-binding proteins or changes in chromatin topology associated with these sites have yet to be elucidated and could be contributing to this inaccessibility.

The FSFS FRA2L in 2p11.2 (Lukusa and Fryns, 2008) was recently reported as the source of the unusual bending of chromosome 2 in metaphase spreads (Garribba et al., 2021). Interestingly, no fragility at 2p11.2 was identified in these experiments, performed in one FXS cell line. Chromosomal bends at this CGG-rich region were observed in the absence of any cellular treatment, together with bending of other chromosomes (chr. 1 and 3), and increased significantly under folate deficient conditions. Folate deficiency also induced chromosome 2 aneuploidy (Garribba et al., 2021). A link between this cytological phenomenon and sister chromatid missegregation is far from being identified, however the role of folate on the stability of CGG-rich regions is confirmed. It appears to be related to differential chromatin compaction and altered DNA replication (effect exacerbated by folate deficiency), which delays the condensation of mitotic chromosomes, allowing for missegregation. Such observations are not new as bending of metaphase chromosomes was first described in 1984 as a change in the direction of the longitudinal axis of the chromosome (45° fold) involving both chromatids (Flejter et al., 1984), and it was further analyzed as a possible indicator of the inactive chromosome X (Van Dyke et al., 1987, 1986). X-chromosome bending was proposed to represent a remnant of the Barr body packaging from the previous interphase or, alternatively, a structural feature that helps to provide continuity to the Barr body from one interphase to the next (Van Dyke et al., 1987, 1986; Munn et al., 1991; Walker et al., 1991; Dietzel et al., 1998). Non-random bends in autosomes have also been described, with higher incidence with increasing length of the chromosomes, and thought to be associated with chromatin compaction as residue of a folded chromosome state in the interphase nucleus (Flejter et al., 1984; Plaja et al., 2004, 2001). More observations on different cell lines are necessary to obtain robust evidence that support a biological role of chromosomal bending and its dependance upon chromatin compaction and accessibility.

Thus, unusual DNA structure formation, in addition to epigenetic factors, can affect fragile site stability. Mechanistically, these secondary structures perturb the elongation of DNA replication *in vitro* and *in vivo* (reviewed in (Kaushal and Freudenreich, 2019) and likely contribute to fragility in this manner*.* Additionally, proteins important for resolving secondary structures, such as helicases and topoisomerases, play a role in the stability of CFSs (Pirzio et al., 2008; Tuduri et al., 2009; Arlt and Glover, 2010; Shah et al., 2010; Murfuni et al., 2012). Aphidicolin-induced replication stress results in uncoupling of the helicase and polymerase activity, leaving up to several kilobases of separated DNA strands that may be prone to forming DNA secondary structures (Dröge et al., 1985; Lönn and Lönn, 1988). Camptothecin, a topoisomerase I inhibitor, reduces breakage at CFSs and in ATR-deficient cells, highlighting a potential role for topoisomerase I in expression of fragile sites (Arlt and Glover, 2010). Furthermore, instability at CFSs was increased upon depletion of the Rev3 subunit of polζ, polη, and possibly polκ (Bergoglio et al., 2013; Bhat et al., 2013), which are DNA polymerases specialized for synthesis through non-canonical DNA structures (Boyer et al., 2013).

The contribution of key epigenetic and DNA structural alterations to fragile site expression have yet to be fully understood. Such *cis* elements are known to influence the susceptibility of these DNA regions to fragility and could ultimately be harnessed to reduce instability. This possibility is particularly relevant for the rare FSFSs, which often exhibit somatic instability and expand in disease contexts*.* Increased knowledge of fragility leading to improved understanding and application to disease biology is a recurring theme for RFSs and highlights the importance of continued research into this often-overlooked cytogenetic phenomenon.

### Replication-transcription collision

Typically, replication and transcriptional activities are temporally coordinated within mammalian cells to avoid problematic collisions. However, many long genes (>800 kb) initiate transcription within G2 and only complete it by late G1/early S phase increasing the chance for collision (Helmrich et al., 2011). More than 80% of human CFSs contain genes larger than 300 kb, which is in striking contrast to the median gene length of ∼20 kb (Le Tallec et al., 2013). Many CFSs harbor exceptionally long genes, which take more than a complete cell cycle to be transcribed, such as the *FHIT* gene (1.5 Mb) at FRA3B (Helmrich et al., 2011). This situation leads to the increased likelihood of collisions between replication and transcriptional machinery, leading to replication fork stalling or collapse and resultant instability (Prado and Aguilera, 2005; Azvolinsky et al., 2009; Merrikh et al., 2011). Supporting this connection is the observation that CFS breaks occur when the implicated genes are transcribed, but not when they are transcriptionally silent (Helmrich et al., 2011). This finding highlights the important role for transcriptional activity in chromosomal fragility.

The conflict between these two metabolic processes is further exacerbated by the propensity of nascent RNA to form RNA:DNA hybrids (R-loops) during transcription (reviewed in (Freudenreich, 2018), particularly in GC-rich regions, which all FSFSs are. (CGG)n expanded loci have been demonstrated to have increased R-loop formation (Reddy et al., 2014, 2011; Groh et al., 2014) and the link between R-loop formation and genomic instability has been a topic of intense study (reviewed in (Freudenreich, 2018; Groh et al., 2014; Santos-Pereira and Aguilera, 2015, p.). Further, knockdown or overexpression of RNase H1 (the primary enzyme responsible for resolving R-loops formed with nascent transcripts), results in increased or decreased expression of fragile sites, respectively (Helmrich et al., 2011). R-loop formation at the FRA16D locus impedes replication and causes replication fork stalling, which is a key aspect of CFS instability (Madireddy et al., 2016). R-loops can also promote the formation of repressive chromatin by altering the local epigenetic landscape (Groh et al., 2014), which also contributes to fragility. The majority of genes present in fragile site regions possess a high propensity for R-loop formation when computationally compared to the rest of the genome (Feng and Chakraborty, 2017). It is important to note that most large genes within the genome remain stable, even if they are able to form R-loops; therefore, gene size *per se* is not sufficient to induce fragility (Le Tallec et al., 2013). A growing awareness of R-loop formations and its connections to genomic instability may yield clues to the relationship between replication, transcription, and fragile sites.

### DNA damage and repair

The contribution of the DNA damage pathway to fragile sites is more extensively studied in relation to CFSs and remains largely unexplored in the field of RFSs. Most notably, the role of the DNA damage response protein kinases ATR (ataxia telangiectasia and Rad3-related), and to a lesser extent ATM (ataxia telangiectasia mutated) have been embedded at the core of the DNA lesion checkpoint response pathways in both common (Casper et al., 2002; Ozeri-Galai et al., 2008), and rare (Kumari et al., 2009) fragile sites. ATR-deficiency causes Seckle syndrome, and cells from these individuals have an increased spontaneous expression of fragile sites (Casper et al., 2004). The Fanconi anemia (FA) repair pathway, which responds to interstrand crosslink (ICL) DNA lesions amongst other functions, plays an integral role in fragile site stability. Cells from FA patients exhibit breakpoints at CFS loci at least 50% of the time (Schoder et al., 2010; Filipović et al., 2016) supporting a connection between DNA repair pathways and fragile sites.

Many other repair proteins are implicated in CFS expression, where their inhibition or knockdown enhances aphidicolin-induced fragility (refer to (Glover et al., 2017) for comprehensive review). Furthermore, proteomic studies of the FRA16D CFS locus revealed that under aphidicolin stress, several repair proteins, such as MSH3, MSH2, XRCC1, WRN, XRCC6, XPC, and CENT2 interact specifically with the locus (Beresova et al., 2016). The complex pathways and overlapping proteins involved in DNA repair of these DNA lesions suggests that other metabolic proteins may be involved in fragile site expression and remain to be identified.

Extensive work by Ian Hickson’s group and others has elucidated a key aspect of the DNA repair pathway involved in processing at CFSs during aphidicolin replicative stress (Chan et al., 2009; Naim et al., 2013; Ying et al., 2013; Minocherhomji and Hickson, 2014; Minocherhomji et al., 2015; Bhowmick et al., 2016): mitotic replicative-stress DNA synthesis, a process known as MiDAS, occurs following the onset of mitosis as a salvage pathway to complete replication of under-replicated loci (Minocherhomji et al., 2015). FANCD2, a member of the FA repair pathway and previously shown to localize to CFS loci (Chan et al., 2009), also colocalizes with >80% of newly replicated DNA foci (Minocherhomji et al., 2015). Furthermore, like fragile sites, this mitotic DNA synthesis occurs at DAPI-negative regions of chromosomes, suggesting that these fragile sites are under-replicated DNA regions, rather than distinct DNA breaks (Minocherhomji et al., 2015). Recent work demonstrated that MiDAS also occurs at the FSFS FRAXA locus (Garribba et al., 2020). While MiDAS processing at CFSs and FRAXA both involve SLX1/4 and POLD3, FRAXA differs in its requirement for RAD51 (but not RAD52 or MUS81-EME1) (Garribba et al., 2020).

Generally, DNA damage and repair at FSFSs remains understudied. ATR, ATM, and Chk1 influence fragile site expression at the FRAXA locus (Kumari et al., 2009), but no other folate-sensitive (CGG)n locus has been examined in regard to the mechanisms related to DNA repair. The depletion of ATR increases fragile site expression in fragile X patient cells, with and without FUdR treatment. ATM inhibition decreases fragile site expression upon FUdR treatment yet, without FUdR treatment, ATM inhibition can increase fragile site expression in fragile X cells (Kumari et al., 2009). Significant headway in the repair-related mechanisms of CFSs could guide studies at RFSs to reveal commonalities and differences in the mechanism of fragile site processing.

## Conclusion

Many key questions remain to be answered in understanding fragility. The recent development of new methods to identify expanded repeats that colocalize with cytogenetically observed, but not molecularly mapped FSFS, has offered tremendous new insight into fragile sites, genome stability, and human disease (Garg et al., 2020; Trost et al., 2020). The convergence of any combination of factors described here could underlie expression of fragile sites at a particular locus, highlighting the complexity of this process. Further, what parameters are required to induce various types of fragile sites, and what commonalities and differences exist in the cellular response to each stressor have yet to be elucidated. Understanding the expression of fragility and the sensitivity of certain loci to replicative stress will be valuable to understanding the mechanisms of genomic instability and countering its effects. Many disease-causing, gene-specific repeat expansions exist at fragile site loci, hinting that mechanisms related to fragility expression could also be contributing factors to DNA expansions at these loci. Understanding the proteins and pathways that contribute to the causes and consequences of these fragile sites could provide useful targets towards therapeutic intervention to stabilize loci and prevent instability at problematic regions linked to a variety of diseases. Prior to the implementation of practical therapeutic steps aimed at improving human health and overcoming disease, it is important to lay down the foundational research to understand the fundamentals of fragile site expression and repeat expansion.
